# Age-structured two-sex HPV transmission dynamics with a carrier state and reactivation

**DOI:** 10.1016/j.idm.2026.10.002

**Published:** 2026-10-20

**Authors:** Siqi Shao, Ting Guo, Shasha Gao

**Affiliations:** a School of Mathematics and Statistics, Jiangxi Normal University, Nanchang, Jiangxi, 330022, China; b Aliyum School of Big Data, Changzhou University, Changzhou, Jiangsu, 213164, China

**Keywords:** Human papillomavirus, Age-structured model, Two-sex model, Viral reactivation

## Abstract

Human papillomavirus (HPV) remains one of the most common sexually transmitted infections worldwide, and its persistence and possible reactivation complicate efforts to understand and control transmission. In particular, the presence of a carrier stage and variation in reactivation across age and sex may play important roles in maintaining HPV prevalence. In this paper, we develop a two-sex Susceptible-Infectious-Carrying-Recovered (SICR) model with *n* discrete age groups to investigate how age-specific and sex-specific reactivation rates affect HPV transmission dynamics and how control measures can be designed accordingly. We derive the basic reproduction number $R_{0}$ and show that it provides a threshold criterion for disease elimination. The existence and stability of both the disease-free equilibrium and the endemic equilibrium are established rigorously. Using epidemiological data from Hangzhou in 2011, we estimate $R_{0}$ to be 2.177. Analytical and numerical results show that viral reactivation plays an important role in sustaining HPV endemicity and highlight the need for age-specific and sex-specific public health strategies. We also find that an excessively low viral reactivation rate is not always beneficial for controlling HPV prevalence. In particular, regulating reactivation alone is insufficient to control HPV transmission effectively, and our results suggest that combined interventions targeting both transmission and reactivation are more effective.

## 1 Introduction

Human papillomavirus (HPV) is a group of highly prevalent sexually transmitted viruses and remains a major public health concern worldwide (WHO, b). Most sexually active individuals, including both women and men, are likely to experience HPV infection or reinfection during their lifetime (He et al., 2025; Sung et al., 2021). Although most HPV infections are asymptomatic and self-limiting, persistent infection with high-risk human papillomavirus (HR-HPV) is the primary cause of several cancers, including cervical, vaginal, vulvar, penile, anal, and oropharyngeal cancers, while benign lesions such as genital warts are also common (Mlynarczyk-Bonikowska & Rudnicka, 2024; Shiels et al., 2015). At present, there is no specific antiviral therapy for HPV, and clinical management mainly relies on lesion removal and immune-based treatment (Mlynarczyk-Bonikowska & Rudnicka, 2024). Among HPV-related diseases, cervical cancer remains one of the leading causes of cancer-related death among women worldwide (Arbyn et al., 2020). According to the World Health Organization, there were approximately 660,000 new cases and 350,000 deaths globally in 2022 (WHO, a). These substantial epidemiological burdens highlight the need for more precise tools to understand and predict HPV transmission dynamics in different populations and to provide a stronger scientific basis for public health interventions.

HPV infection also presents an important epidemiological challenge. Although most infections are cleared spontaneously by the immune system, with an average clearance time of approximately 13.4 months for low-risk HPV types and 16.3 months for high-risk types (Richardson et al., 2003), HPV still causes a large disease burden because a subset of infections persists and may progress through distinct pathogenic pathways. Persistent infection is particularly important in HPV-related carcinogenesis (Huber et al., 2021). It has been reported that 4–10% of middle-aged women remain persistent carriers, representing a high-risk population for cervical cancer (Bosch et al., 2013). Moreover, the 20–40 year lag between the peak of HPV infection in younger populations and the occurrence of cervical cancer in middle-aged women underscores the long-term tumorigenic role of persistent infection (Coelho et al., 2015). In addition, increasing evidence suggests that distinguishing viral clearance from long-term carriage, as well as accounting for viral reactivation, is important for accurately characterizing HPV transmission dynamics (Gravitt, 2012; Maglennon et al., 2014; Malagón et al., 2022). Therefore, incorporating transitions between a carrier state and active infection may provide a more realistic description of the long-term persistence and prevalence of HPV.

Age-structured mathematical models have become important tools for analyzing HPV epidemiology and evaluating prevention and control strategies. By quantifying biological characteristics and behavioral differences across age groups, such models can capture essential heterogeneity in transmission and disease progression. Many studies have used age-structured models to investigate HPV transmission dynamics, and their simulation results are broadly consistent with observed epidemiological patterns (Akimenko & Adi-Kusumo, 2021; Malagón et al., 2018; Peng et al., 2018; Sari et al., 2022; Saxena et al., 2024). For example, Saxena et al. suggested that increasing vaccination initiation rates among 9-year-olds could reduce disparities across subgroups in the time required to achieve vaccination completion targets (Saxena et al., 2024). By integrating age structure with multistage disease progression, Sari et al. proposed an age-stratified prevention strategy in which HPV vaccination is prioritized for individuals aged 15–25 years to reduce infection rates, cervical cancer screening is strengthened for those aged 35 years and above for early lesion detection, and screening frequency is optimized for individuals aged 60 years and above to reduce progression risk (Sari et al., 2022). Malagón et al. found that excluding women with hysterectomies could underestimate cervical cancer incidence by up to 71% among women aged 80–84 years (Malagón et al., 2018). These studies illustrate the importance of age structure in understanding HPV burden and designing targeted interventions.

Two-sex models have also played an important role in studying HPV transmission between females and males. Some works have focused primarily on the mathematical analysis and numerical simulation of HPV transmission models (Azevedo et al., 2019; Malik et al., 2013; Omame et al., 2018; Sharomi & Malik, 2017; Ziyadi, 2017), while many others have incorporated vaccination strategies into sex-structured modeling frameworks (Cody et al., 2021; Gao et al., 2022; Ren et al., 2025; Saldana et al., 2020; Zhong et al., 2021). For example, Ren et al. found that vaccination should be prioritized for 14-year-old females, followed by 14-year-old males (Ren et al., 2025). In our previous work, we found that vaccines should first be allocated to the sex with the smaller recruitment rate, and that, compared with allocating the same number of vaccine doses annually, allocating more doses in the early stage and fewer in the later stage could reduce both the time and the total number of doses needed to control prevalence below a prescribed threshold (Gao et al., 2022). Other studies have suggested that girls-only vaccination programs may be more cost-effective (Cody et al., 2021; Saldana et al., 2020). In general, two-sex models can explicitly account for differences between females and males and therefore describe transmission chains driven by sexual partnerships more realistically than models that do not distinguish sex (Kretzschmar & Heijne, 2017).

Despite the substantial literature on HPV transmission modeling, relatively few studies have simultaneously incorporated age structure, two-sex transmission, and viral reactivation from a carrier state within a unified framework. In particular, existing HPV models often neglect the combined effects of age-specific and sex-specific reactivation heterogeneity. To address this gap, we formulate a two-sex mathematical model with *n* discrete age groups to investigate how variation in reactivation rates across age and sex influences the transmission dynamics of HPV and helps sustain endemic infection. Our model integrates these features into a single framework, allowing us to study the long-term persistence of HPV and to evaluate targeted intervention strategies. Specifically, we aim to address the following questions: (1) how do viral reactivation rates across different age groups and sexes affect the transmission dynamics of HPV? and (2) what intervention measures are most effective for controlling HPV prevalence? This work provides a theoretical framework for understanding the coupled effects of age structure, sex structure, and reactivation on HPV transmission, and offers a useful tool for evaluating the potential impact of public health interventions. The model is formulated in Section 2 and analyzed in Section 3. Numerical simulations are presented in Section 4, followed by discussion in Section 5.

## 2 Model formulation

In this section, we formulate an SICR model with *n* discrete age groups that incorporates two-sex transmission and viral reactivation to study HPV transmission in a heterosexually active population. Subscripts 1, …, *n* are used to denote the *n* age groups. In each age group, the population is divided into two subpopulations: heterosexual females and heterosexual males, referred to simply as females and males below. We use superscripts *f* and *m* to denote these two groups, respectively. Within each subgroup, the population is further divided into four epidemiological classes: susceptible individuals $\left(S_{i}^{g}\left(t\right)\right)$, infectious individuals $\left(I_{i}^{g}\left(t\right)\right)$, virus carriers $\left(C_{i}^{g}\left(t\right)\right)$, and recovered individuals $\left(R_{i}^{g}\left(t\right)\right)$, where *g* ∈ {*f*, *m*} and *i* ∈ {1, …, *n*}.

We assume that individuals within each compartment mix homogeneously, so that all individuals in the same compartment experience the same average force of infection. Since recovery from HPV infection does not acquire lifelong immunity, recovered individuals may lose protection and become susceptible. In addition, viral carriers and actively infected individuals are assumed to have different levels of infectiousness to susceptible individuals, and their contributions to the force of infection are modeled separately.

We denote by *δ*_*i*_(*i* = 1, 2, …, *n*) the adjustment parameter for the *i*th age group, where *δ*_1_ = 1 and *δ*_*i*_ = 0 for *i* = 2, …, *n*, so that all newly recruited individuals enter the first age group. We denote by $c_{ij}^{fm}$ the contact rate between females in age group *i* and males in age group *j*, by *β*_*mf*_ the probability that a male infects a female, and by *σ* the attenuation coefficient measuring the infectivity of virus carriers relative to that of actively infectious individuals (typically *σ* < 1). Similarly, $c_{ij}^{mf}$ and *β*_*fm*_ have analogous meanings. We denote by $a_{i-1}^{g}$ the transition rate from the (*i* − 1)th age group to the *i*th age group for sex *g*(*i* ≥ 1). Under the closed-boundary assumption for the age classes, we set $a_{0}^{g}=a_{n}^{g}=0$. The model (1) is given as follows. A schematic diagram of the model is shown in Fig. 1, and detailed descriptions of the variables and parameters are provided in Table 1, Table 2.

(1) $$\left\{\begin{array}{ll} \frac{dS_{i}^{g}}{dt} & =\Lambda^{g}\delta_{i}+\omega_{i}^{g}R_{i}^{g}-\lambda_{i}^{g}\left(t\right)S_{i}^{g}-\left(a_{i}^{g}+\mu_{i}^{g}\right)S_{i}^{g}+a_{i-1}^{g}S_{i-1}^{g},\\ \frac{dI_{i}^{g}}{dt} & =\lambda_{i}^{g}\left(t\right)S_{i}^{g}+\alpha_{i}^{g}C_{i}^{g}-\left(\gamma_{i}^{g}+a_{i}^{g}+\mu_{i}^{g}\right)I_{i}^{g}+a_{i-1}^{g}I_{i-1}^{g},\\ \frac{dC_{i}^{g}}{dt} & =\left(1-p\right)\gamma_{i}^{g}I_{i}^{g}-\left(\alpha_{i}^{g}+a_{i}^{g}+\mu_{i}^{g}\right)C_{i}^{g}+a_{i-1}^{g}C_{i-1}^{g},\\ \frac{dR_{i}^{g}}{dt} & =p\gamma_{i}^{g}I_{i}^{g}-\left(\omega_{i}^{g}+a_{i}^{g}+\mu_{i}^{g}\right)R_{i}^{g}+a_{i-1}^{g}R_{i-1}^{g}, \end{array}\right)$$

with

$$S_{i}^{g}\left(0\right)=S_{i}^{g0},I_{i}^{g}\left(0\right)=I_{i}^{g0},C_{i}^{g}\left(0\right)=C_{i}^{g0},R_{i}^{g}\left(0\right)=R_{i}^{g0}.$$

**Fig. 1:**
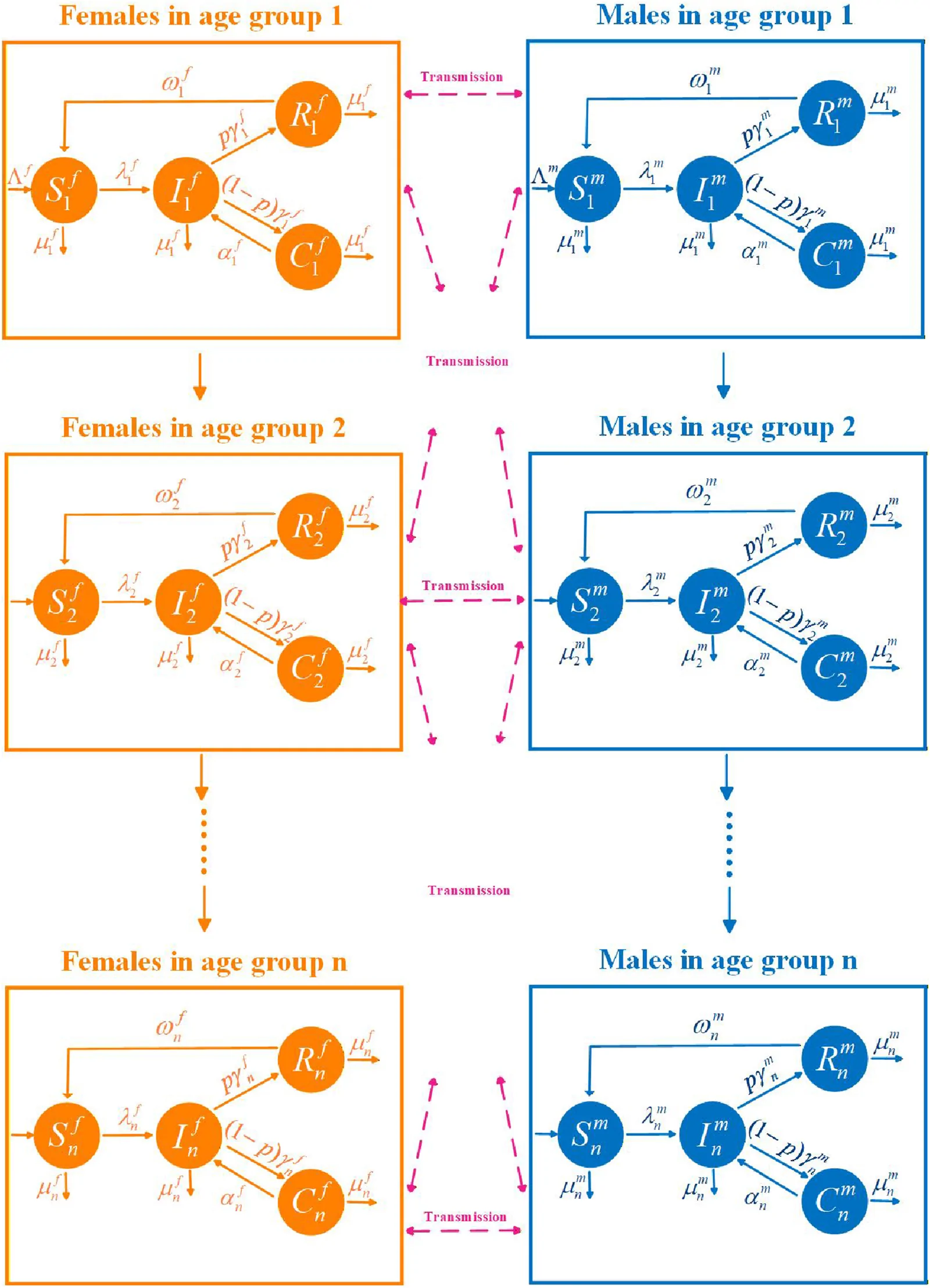
Flow diagram of the discrete *n*-age-group SICR model (1). In each age group, the population is divided into four classes: susceptible, infectious, carrier, and recovered individuals, denoted by *S*, *I*, *C*, and *R*, respectively. Transmission occurs between females and males both within the same age group and across different age groups. Descriptions of the variables and parameters are given in Table 1, Table 2, respectively.

**Table 1:** Description of the state variables in model (1).

Variable	Description
$S_{i}^{g}$	Susceptible individuals in the *i*th age group of sex *g*
$I_{i}^{g}$	Infectious individuals in the *i*th age group of sex *g*
$C_{i}^{g}$	Virus carriers in the *i*th age group of sex *g*
$R_{i}^{g}$	Recovered individuals in the *i*th age group of sex *g*

**Table 2:** Description of the parameters in model (1).

Parameter	Description
Λ^*g*^	Recruitment rate of individuals of sex *g*
$\lambda_{i}^{g}$	Force of infection for individuals in the *i*th age group of sex *g*
*δ*_*i*_	Adjustment parameter for recruitment into the *i*th age group (*δ*_1_ = 1, *δ*_*i*_ = 0 for *i* ≥ 2)
$c_{ij}^{g_{1}g_{2}}$	Number of sexual contacts per unit time that an individual of sex *g*_1_ in the *i*th age group has with individuals of sex *g*_2_ in the *j*th age group (*g*_1_, *g*_2_ ∈ {*f*, *m*}, *g*_1_ ≠ *g*_2_)
$\beta_{g_{1}g_{2}}$	Probability of virus transmission from an individual of sex *g*_1_ to a sexual partner of sex *g*_2_ per contact
$\omega_{i}^{g}$	Waning rate of immunity for individuals in the *i*th age group of sex *g*
$a_{i-1}^{g}$	Transition rate from the (*i* − 1)^th^ age group to the *i*th age group for sex *g*(*i* ≥ 1)
$\mu_{j}^{g}$	Natural death rate for individuals in the *j*th age group of sex *g*
$\alpha_{i}^{g}$	Reactivation rate from $C_{i}^{g}$ to $I_{i}^{g}$
$\gamma_{i}^{g}$	Recovery rate of infectious individuals in the *i*th age group of sex *g*
*p*	Probability of complete viral clearance
*σ*	Relative infectivity of virus carriers compared with infectious individuals

The forces of infection for females and males are given by

$$\lambda_{i}^{f}\left(t\right)=\overset{n}{\underset{j=1}{\sum }}c_{ij}^{fm}\frac{\beta_{mf}\cdot \left(I_{j}^{m}\left(t\right)+\sigma \cdot C_{j}^{m}\left(t\right)\right)}{N_{j}^{m}\left(t\right)},\;\lambda_{i}^{m}\left(t\right)=\overset{n}{\underset{j=1}{\sum }}c_{ij}^{mf}\frac{\beta_{fm}\cdot \left(I_{j}^{f}\left(t\right)+\sigma \cdot C_{j}^{f}\left(t\right)\right)}{N_{j}^{f}\left(t\right)},$$

where $N_{i}^{g}=S_{i}^{g}+I_{i}^{g}+C_{i}^{g}+R_{i}^{g}$ with *g* ∈ {*f*, *m*} and *i* ∈ {1, …, *n*} denotes the total population of sex *g* in age group *i*. The total population is denoted by

$$N=N^{f}+N^{m}=\underset{g\in \left\{f,m\right\}}{\sum }\overset{n}{\underset{j=1}{\sum }}N_{j}^{g}=\underset{g\in \left\{f,m\right\}}{\sum }\overset{n}{\underset{j=1}{\sum }}\left(S_{j}^{g}+I_{j}^{g}+C_{j}^{g}+R_{j}^{g}\right).$$

By summing $S_{i}^{g}$, $I_{i}^{g}$, $C_{i}^{g}$, and $R_{i}^{g}$ in system (1), we obtain

$$\frac{dN^{g}}{dt}=\Lambda^{g}-\overset{n}{\underset{j=1}{\sum }}\mu_{i}^{g}N_{i}^{g}.$$

According to the Intermediate Value Theorem, there exists a $\mu^{g}\in \left[\mu_{\text{min}}^{g},\mu_{\text{max}}^{g}\right]$ such that

$$\frac{dN^{g}}{dt}=\Lambda^{g}-\overset{n}{\underset{j=1}{\sum }}\mu_{i}^{g}N_{i}^{g}=\Lambda^{g}-\mu^{g}N^{g},\;g\in \left\{f,m\right\},$$

where

$$\mu_{\text{min}}^{g}=\min\left\{\mu_{i}^{g}\right\},\;\mu_{\text{max}}^{g}=\max\left\{\mu_{i}^{g}\right\},\;i=1,\ldots ,n.$$

We define the feasible region of model (1) by

$$\begin{array}{ll} D= & \left\{\left(S_{1}^{f},\ldots ,S_{n}^{f},I_{1}^{f},\ldots ,I_{n}^{f},C_{1}^{f},\ldots ,C_{n}^{f},R_{1}^{f},\ldots ,R_{n}^{f},S_{1}^{m},\ldots ,S_{n}^{m},I_{1}^{m},\ldots ,I_{n}^{m},C_{1}^{m},\ldots ,C_{n}^{m},\right)\right)\\ \left(R_{1}^{m},\ldots ,R_{n}^{m}\right) & \in R_{+}^{8n}:\overset{n}{\underset{j=1}{\sum }}\left(\left(S_{j}^{g}+I_{j}^{g}+C_{j}^{g}+R_{j}^{g}\right)\leq \frac{\Lambda^{g}}{\mu^{g}},\;g\in \left\{f,m\right\}\right\}. \end{array}$$

It can be verified that *D* is positively invariant under system (1). Therefore, the model is both epidemiologically and mathematically well posed (see Appendix A).

## 3 Mathematical analysis

### 3.1 The disease-free equilibrium

System (1) always admits a unique disease-free equilibrium (DFE). At the disease-free equilibrium,

$$I_{i}^{g}=0,\;C_{i}^{g}=0,$$

$$\lambda_{i}^{f}\left(t\right)=\overset{n}{\underset{j=1}{\sum }}c_{ij}^{fm}\frac{\beta_{mf}\left(I_{j}^{m}\left(t\right)+\sigma \cdot C_{j}^{m}\left(t\right)\right)}{N_{j}^{m}\left(t\right)}=0;\lambda_{i}^{m}\left(t\right)=\overset{n}{\underset{j=1}{\sum }}c_{ij}^{mf}\frac{\beta_{fm}\left(I_{j}^{f}\left(t\right)+\sigma \cdot C_{j}^{f}\left(t\right)\right)}{N_{j}^{f}\left(t\right)}=0,$$

and all state variables remain constant over time. Equivalently, all derivatives in system (1) are equal to zero:

(2) $$\left\{\begin{array}{ll} 0 & =\Lambda^{g}\delta_{i}+\omega_{i}^{g}R_{i}^{g}-\lambda_{i}^{g}\left(t\right)S_{i}^{g}-\left(a_{i}^{g}+\mu_{i}^{g}\right)S_{i}^{g}+a_{i-1}^{g}S_{i-1}^{g},\\ 0 & =\lambda_{i}^{g}\left(t\right)S_{i}^{g}+\alpha_{i}^{g}C_{i}^{g}-\left(\gamma_{i}^{g}+a_{i}^{g}+\mu_{i}^{g}\right)I_{i}^{g}+a_{i-1}^{g}I_{i-1}^{g},\\ 0 & =\left(1-p\right)\gamma_{i}^{g}I_{i}^{g}-\left(\alpha_{i}^{g}+a_{i}^{g}+\mu_{i}^{g}\right)C_{i}^{g}+a_{i-1}^{g}C_{i-1}^{g},\\ 0 & =p\gamma_{i}^{g}I_{i}^{g}-\left(\omega_{i}^{g}+a_{i}^{g}+\mu_{i}^{g}\right)R_{i}^{g}+a_{i-1}^{g}R_{i-1}^{g}. \end{array}\right)$$

System (2) can be reduced to

(3) $$\left\{\begin{array}{ll} 0 & =\Lambda^{g}\delta_{i}+\omega_{i}^{g}R_{i}^{g}-\left(a_{i}^{g}+\mu_{i}^{g}\right)S_{i}^{g}+a_{i-1}^{g}S_{i-1}^{g},\\ 0 & =-\left(\omega_{i}^{g}+a_{i}^{g}+\mu_{i}^{g}\right)R_{i}^{g}+a_{i-1}^{g}R_{i-1}^{g}. \end{array}\right)$$

From the second equation of system (3), we obtain

$$\left(\omega_{i}^{g}+a_{i}^{g}+\mu_{i}^{g}\right)R_{i}^{g}=a_{i-1}^{g}R_{i-1}^{g}.$$

Since

$$a_{0}^{g}=0,$$

it follows that

$$R_{1}^{g}=0.$$

By mathematical induction, we then obtain

$$R_{i}^{g}=0,\;i=1,2,\ldots ,n.$$

Substituting this into the first equation of system (3), we get

$$0=\Lambda^{g}\delta_{i}-\left(a_{i}^{g}+\mu_{i}^{g}\right)S_{i}^{g}+a_{i-1}^{g}S_{i-1}^{g}.$$

It remains to solve this linear recurrence relation for $S_{i}^{g}$.

For the first age group (*i* = 1),

$$\delta_{1}=1,\;a_{0}^{g}S_{0}^{g}=0,\;0=\Lambda^{g}-\left(a_{1}^{g}+\mu_{1}^{g}\right)S_{1}^{g},$$

which gives

$$S_{1}^{g0}=\frac{\Lambda^{g}}{a_{1}^{g}+\mu_{1}^{g}}.$$

For the subsequent age groups (*i* > 1),

$$\delta_{i}=0,\;0=-\left(a_{i}^{g}+\mu_{i}^{g}\right)S_{i}^{g}+a_{i-1}^{g}S_{i-1}^{g},$$

which yields

$$S_{i}^{g0}=\frac{a_{i-1}^{g}}{a_{i}^{g}+\mu_{i}^{g}}S_{i-1}^{g0}.$$

Therefore, the disease-free equilibrium of this sex- and age-structured SICR model, denoted by *E*^0^, is given by

$$E^{0}={\left(S_{1}^{f0},\ldots ,S_{n}^{f0},S_{1}^{m0},\ldots ,S_{n}^{m0},0,\ldots ,0\right)}_{1\times 8n}.$$

### 3.2 The basic reproduction number

The basic reproduction number, denoted by $R_{0}$, is defined as the expected number of secondary HPV infections generated by a typical infectious individual over the course of the infectious period in a fully susceptible population. Using the next-generation matrix approach (van den Driessche & Watmough, 2002), we consider the infected-state vector

$$x=\left(I_{1}^{f},\ldots ,I_{n}^{f},C_{1}^{f},\ldots ,C_{n}^{f},I_{1}^{m},\ldots ,I_{n}^{m},C_{1}^{m},\ldots ,C_{n}^{m}\right).$$

It follows that

$$\dot{x}=F\left(x\right)-V\left(x\right),$$

where

$$F\left(x\right)={\left(\begin{array}{l} \lambda_{1}^{f}\left(t\right)S_{1}^{f}\\ \vdots \\ \lambda_{n}^{f}\left(t\right)S_{1}^{f}\\ 0\\ \vdots \\ 0\\ \lambda_{1}^{m}\left(t\right)S_{1}^{m}\\ \vdots \\ \lambda_{n}^{m}\left(t\right)S_{1}^{m}\\ 0\\ \vdots \\ 0 \end{array}\right)}_{4n\times 1},V\left(x\right)={\left(\begin{array}{l} -\alpha_{1}^{f}C_{1}^{f}+\left(\gamma_{1}^{f}+a_{1}^{f}+\mu_{1}^{f}\right)I_{1}^{f}-a_{0}^{f}I_{0}^{f}\\ \vdots \\ -\alpha_{n}^{f}C_{n}^{f}+\left(\gamma_{n}^{f}+a_{n}^{f}+\mu_{n}^{f}\right)I_{n}^{f}-a_{n-1}^{f}I_{n-1}^{f}\\ -\left(1-p\right)\gamma_{1}^{f}I_{1}^{f}+\left(\alpha_{1}^{f}+a_{1}^{f}+\mu_{1}^{f}\right)C_{1}^{f}-a_{0}^{f}C_{0}^{f}\\ \vdots \\ -\left(1-p\right)\gamma_{n}^{f}I_{n}^{f}+\left(\alpha_{n}^{f}+a_{n}^{f}+\mu_{n}^{f}\right)C_{n}^{f}-a_{n-1}^{f}C_{n-1}^{f}\\ -\alpha_{1}^{m}C_{1}^{m}+\left(\gamma_{1}^{m}+a_{1}^{m}+\mu_{1}^{m}\right)I_{1}^{m}-a_{0}^{m}I_{0}^{m}\\ \vdots \\ -\alpha_{n}^{m}C_{n}^{m}+\left(\gamma_{n}^{m}+a_{n}^{m}+\mu_{n}^{m}\right)I_{n}^{m}-a_{n-1}^{m}I_{n-1}^{m}\\ -\left(1-p\right)\gamma_{1}^{m}I_{1}^{m}+\left(\alpha_{1}^{m}+a_{1}^{m}+\mu_{1}^{m}\right)C_{1}^{m}-a_{0}^{m}C_{0}^{m}\\ \vdots \\ -\left(1-p\right)\gamma_{n}^{m}I_{n}^{m}+\left(\alpha_{n}^{m}+a_{n}^{m}+\mu_{n}^{m}\right)C_{n}^{m}-a_{n-1}^{m}C_{n-1}^{m} \end{array}\right)}_{4n\times 1}.$$

Taking the Jacobian matrices of F and V at the DFE *E*^0^, we obtain

$$F\overset{△}{=}\left(\begin{array}{ll} 0 & F_{fm}\\ F_{mf} & 0 \end{array}\right)={\left(\begin{array}{llll} 0 & 0 & F_{13} & F_{14}\\ 0 & 0 & 0 & 0\\ F_{31} & F_{32} & 0 & 0\\ 0 & 0 & 0 & 0 \end{array}\right)}_{4n\times 4n},\;V={\left(\begin{array}{ll} V^{f} & 0\\ 0 & V^{m} \end{array}\right)}_{4n\times 4n},$$

where

$$F_{13}={\left(\begin{array}{llll} \frac{c_{11}^{fm}\beta_{mf}}{N_{1}^{m0}}S_{1}^{f0} & \frac{c_{12}^{fm}\beta_{mf}}{N_{2}^{m0}}S_{1}^{f0} & \ldots & \frac{c_{1n}^{fm}\beta_{mf}}{N_{n}^{m0}}S_{1}^{f0}\\ \frac{c_{21}^{fm}\beta_{mf}}{N_{1}^{m0}}S_{2}^{f0} & \frac{c_{22}^{fm}\beta_{mf}}{N_{2}^{m0}}S_{2}^{f0} & \ldots & \frac{c_{2n}^{fm}\beta_{mf}}{N_{n}^{m0}}S_{2}^{f0}\\ \vdots & \vdots & \ddots & \vdots \\ \frac{c_{n1}^{fm}\beta_{mf}}{N_{1}^{m0}}S_{n}^{f0} & \frac{c_{n2}^{fm}\beta_{mf}}{N_{2}^{m0}}S_{n}^{f0} & \ldots & \frac{c_{nn}^{fm}\beta_{mf}}{N_{n}^{m0}}S_{n}^{f0} \end{array}\right)}_{n\times n},$$

$$F_{31}={\left(\begin{array}{llll} \frac{c_{11}^{mf}\beta_{fm}}{N_{1}^{f0}}S_{1}^{m0} & \frac{c_{12}^{mf}\beta_{fm}}{N_{2}^{f0}}S_{1}^{m0} & \ldots & \frac{c_{1n}^{mf}\beta_{fm}}{N_{n}^{f0}}S_{1}^{m0}\\ \frac{c_{21}^{mf}\beta_{fm}}{N_{1}^{f0}}S_{2}^{m0} & \frac{c_{22}^{mf}\beta_{fm}}{N_{2}^{f0}}S_{2}^{m0} & \ldots & \frac{c_{2n}^{mf}\beta_{fm}}{N_{n}^{f0}}S_{2}^{m0}\\ \vdots & \vdots & \ddots & \vdots \\ \frac{c_{n1}^{mf}\beta_{fm}}{N_{1}^{f0}}S_{n}^{m0} & \frac{c_{n2}^{mf}\beta_{fm}}{N_{2}^{f0}}S_{n}^{m0} & \ldots & \frac{c_{nn}^{mf}\beta_{fm}}{N_{n}^{f0}}S_{n}^{m0} \end{array}\right)}_{n\times n},$$

$$F_{14}=\sigma F_{13},\;F_{32}=\sigma F_{31}.$$

$$V^{g}={\left(\begin{array}{ll} V_{II}^{g} & V_{IC}^{g}\\ V_{CI}^{g} & V_{CC}^{g} \end{array}\right)}_{2n\times 2n}\text{with}\;g\in \left\{f,m\right\},$$

where

$$V_{II}^{g}={\left(\begin{array}{llllll} \gamma_{1}^{g}+a_{1}^{g}+\mu_{1}^{g} & 0 & \ldots & 0 & 0 & 0\\ -a_{1}^{g} & \gamma_{2}^{g}+a_{2}^{g}+\mu_{2}^{g} & \ldots & 0 & 0 & 0\\ 0 & -a_{2}^{g} & \ldots & 0 & 0 & 0\\ 0 & 0 & \ldots & 0 & 0 & 0\\ \vdots & \vdots & \ddots & \vdots & \vdots & \vdots \\ 0 & 0 & \ldots & 0 & -a_{n-1}^{g} & \gamma_{n}^{g}+a_{n}^{g}+\mu_{n}^{g} \end{array}\right)}_{n\times n},$$

$$\;V_{IC}^{g}={\left(\begin{array}{lll} -\alpha_{1}^{g} & & \\ & \ddots & \\ & & -\alpha_{n}^{g} \end{array}\right)}_{n\times n},$$

$$\;V_{CI}^{g}={\left(\begin{array}{lll} -\left(1-p\right)\gamma_{1}^{g} & & \\ & \ddots & \\ & & -\left(1-p\right)\gamma_{n}^{g} \end{array}\right)}_{n\times n},$$

$$V_{CC}^{g}={\left(\begin{array}{lllll} \alpha_{1}^{g}+a_{1}^{g}+\mu_{1}^{g} & 0 & \ldots & 0 & 0\\ -a_{1}^{g} & \alpha_{2}^{g}+a_{2}^{g}+\mu_{2}^{g} & \ldots & 0 & 0\\ 0 & -a_{2}^{g} & \ldots & 0 & 0\\ \vdots & \vdots & \ddots & \vdots & \vdots \\ 0 & 0 & \ldots & -a_{n-1}^{g} & \alpha_{n}^{g}+a_{n}^{g}+\mu_{n}^{g} \end{array}\right)}_{n\times n}.$$

Then the next-generation matrix *FV*^−1^ can be written as

$$K\overset{△}{=}FV^{-1}=\left(\begin{array}{ll} 0 & F_{fm}\\ F_{mf} & 0 \end{array}\right)\left(\begin{array}{ll} {\left(V^{f}\right)}^{-1} & 0\\ 0 & {\left(V^{m}\right)}^{-1} \end{array}\right)=\left(\begin{array}{ll} 0 & K_{fm}\\ K_{mf} & 0 \end{array}\right),$$

where

$${\left(V^{g}\right)}^{-1}=\left(\begin{array}{ll} v_{11}^{g} & v_{12}^{g}\\ v_{21}^{g} & v_{22}^{g} \end{array}\right),\;g\in \left\{f,m\right\},$$

$$K_{fm}=F_{fm}{\left(V^{m}\right)}^{-1},\;K_{mf}=F_{mf}{\left(V^{f}\right)}^{-1},$$

$$v_{11}^{g}={\left(V_{II}^{g}\right)}^{-1}+{\left(V_{II}^{g}\right)}^{-1}V_{IC}^{g}{\left[V_{CC}^{g}-V_{CI}^{g}{\left(V_{II}^{g}\right)}^{-1}V_{IC}^{g}\right]}^{-1}V_{CI}^{g}{\left(V_{II}^{g}\right)}^{-1},$$

$$v_{12}^{g}=-{\left(V_{II}^{g}\right)}^{-1}V_{IC}^{g}{\left[V_{CC}^{g}-V_{CI}^{g}{\left(V_{II}^{g}\right)}^{-1}V_{IC}^{g}\right]}^{-1},$$

$$v_{21}^{g}=-{\left[V_{CC}^{g}-V_{CI}^{g}{\left(V_{II}^{g}\right)}^{-1}V_{IC}^{g}\right]}^{-1}V_{CI}^{g}{\left(V_{II}^{g}\right)}^{-1},$$

$$v_{22}^{g}={\left[V_{CC}^{g}-V_{CI}^{g}{\left(V_{II}^{g}\right)}^{-1}V_{IC}^{g}\right]}^{-1}.$$

Remark 1 The 2*n* × 2*n* matrix *K*_*fm*_ is the next-generation operator from males to females. It describes the total number of newly infected females generated by an initial infected male, together with the distribution of these newly infected individuals across age groups and infection states (*I* and *C*), over all possible infection states and age groups of the infective male. The matrix *K*_*mf*_ has a similar interpretation.

Our goal is to determine $R_{0}=\rho \left(K\right)$. To this end, we analyze the eigenvalues of the matrix *K*. If $v={\left(v_{f},\;v_{m}\right)}^{T}$ is an eigenvector of *K* associated with eigenvalue *μ*, then

$$\left(\begin{array}{ll} 0 & K_{fm}\\ K_{mf} & 0 \end{array}\right)\left(\begin{array}{l} v_{f}\\ v_{m} \end{array}\right)=\mu \left(\begin{array}{l} v_{f}\\ v_{m} \end{array}\right).$$

This can be decomposed into the two equations

$$\left\{\begin{array}{l} K_{fm}v_{m}=\mu v_{f},\\ K_{mf}v_{f}=\mu v_{m}. \end{array}\right)$$

Substituting the first equation into the second yields

$$K_{mf}K_{fm}v_{m}=\mu^{2}v_{m}.$$

Similarly, we obtain

$$K_{fm}K_{mf}v_{f}=\mu^{2}v_{f}.$$

This shows that *μ*^2^ is an eigenvalue of both *K*_*mf*_*K*_*fm*_ and *K*_*fm*_*K*_*mf*_. Conversely, if *λ* ≥ 0 is an eigenvalue of either *K*_*mf*_*K*_*fm*_ or *K*_*fm*_*K*_*mf*_, then both $+\sqrt{\lambda }$ and $-\sqrt{\lambda }$ are eigenvalues of the matrix *K*. Therefore, we conclude that

$${\left(\rho \left(K\right)\right)}^{2}=\rho \left(K_{mf}K_{fm}\right)=\rho \left(K_{fm}K_{mf}\right).$$

Hence,

$$R_{0}=\rho \left(FV^{-1}\right)=\rho \left(K\right)=\sqrt{\rho \left(K_{mf}K_{fm}\right)}=\sqrt{\rho \left(K_{fm}K_{mf}\right)}.$$

We denote the two-generation cycle matrices for females and males by

$$R_{f}=K_{fm}K_{mf}\;\text{and}\;R_{m}=K_{mf}K_{fm}.$$

Remark 2 In this model, for an infected female to generate newly infected females, transmission must pass through males, forming an *f* → *m* → *f* cycle. Hence, $R_{f}$ describes the complete two-generation transmission cycle starting from an infected female, passing through one generation of transmission in males, and returning to the female population. The matrix $R_{m}$ has a similar interpretation.

It follows that

$$\rho \left(R_{f}\right)=\rho \left(K_{fm}K_{mf}\right)=\rho \left(K_{mf}K_{fm}\right)=\rho \left(R_{m}\right),$$

and

$$R_{0}=\sqrt{\rho \left(K_{mf}K_{fm}\right)}={\left(\rho \left(R_{f}\right)\rho \left(R_{m}\right)\right)}^{\frac{1}{4}}.$$

We note that $R_{0}$ is the threshold quantity for HPV transmission and measures the geometric mean number of secondary infections generated by a typical infected individual introduced into a fully susceptible population. Next, we consider two special cases.

Case 1 Basic reproduction number within one age group

We can obtain the basic reproduction number (see Appendix B) for one age group:

$$R_{0}=\sqrt{R_{0}^{f1}R_{0}^{m1}},$$

where

$$R_{0}^{m1}=c_{11}^{fm}\frac{\beta_{mf}}{N_{1}^{m}\left(t\right)}S_{1}^{f}\frac{\alpha_{1}^{m}+\mu_{1}^{m}}{D^{m}}+\sigma c_{11}^{fm}\frac{\beta_{mf}}{N_{1}^{m}\left(t\right)}S_{1}^{f}\frac{\left(1-p\right)\gamma_{1}^{m}}{D^{m}},$$

$$R_{0}^{f1}=c_{11}^{mf}\frac{\beta_{fm}}{N_{1}^{f}\left(t\right)}S_{1}^{m}\frac{\alpha_{1}^{f}+\mu_{1}^{f}}{D^{f}}+\sigma c_{11}^{mf}\frac{\beta_{fm}}{N_{1}^{f}\left(t\right)}S_{1}^{m}\frac{\left(1-p\right)\gamma_{1}^{f}}{D^{f}}.$$

with

$$D^{g}=\frac{1}{\left(\gamma_{1}^{g}+\mu_{1}^{g}\right)\left(\alpha_{1}^{g}+\mu_{1}^{g}\right)-\left(1-p\right)\gamma_{1}^{g}\alpha_{1}^{g}}=\frac{1}{\left(\gamma_{1}^{g}+\mu_{1}^{g}\right)\left(\alpha_{1}^{g}+\mu_{1}^{g}\right)}\left[\frac{1}{1-\frac{\left(1-p\right)\gamma_{1}^{g}\alpha_{1}^{g}}{\left(\gamma_{1}^{g}+\mu_{1}^{g}\right)\left(\alpha_{1}^{g}+\mu_{1}^{g}\right)}}\right],g\in \left\{f,m\right\}.$$

It can be seen that $R_{0}^{m1}$ can be decomposed into two components, namely

$$c_{11}^{fm}\frac{\beta_{mf}}{N_{1}^{m}\left(t\right)}S_{1}^{f}\frac{\alpha_{1}^{m}+\mu_{1}^{m}}{D^{m}}=\frac{c_{11}^{fm}\beta_{mf}\frac{S_{1}^{f}}{N_{1}^{m}\left(t\right)}}{\gamma_{1}^{m}+\mu_{1}^{m}}\cdot \overset{\infty }{\underset{n=0}{\sum }}{\left[\frac{\left(1-p\right)\gamma_{1}^{m}\alpha_{1}^{m}}{\left(\gamma_{1}^{m}+\mu_{1}^{m}\right)\left(\alpha_{1}^{m}+\mu_{1}^{m}\right)}\right]}^{n},$$

and

$$\sigma c_{11}^{fm}\frac{\beta_{mf}}{N_{1}^{m}\left(t\right)}S_{1}^{f}\frac{\left(1-p\right)\gamma_{1}^{m}}{D^{m}}=\frac{\sigma c_{11}^{fm}\beta_{mf}\frac{S_{1}^{f}}{N_{1}^{m}\left(t\right)}\left(1-p\right)\gamma_{1}^{m}}{\left(\gamma_{1}^{m}+\mu_{1}^{m}\right)\left(\alpha_{1}^{m}+\mu_{1}^{m}\right)}\cdot \overset{\infty }{\underset{n=0}{\sum }}{\left[\frac{\left(1-p\right)\gamma_{1}^{m}\alpha_{1}^{m}}{\left(\gamma_{1}^{m}+\mu_{1}^{m}\right)\left(\alpha_{1}^{m}+\mu_{1}^{m}\right)}\right]}^{n},$$

where $c_{11}^{fm}\beta_{mf}\frac{S_{1}^{f}}{N_{1}^{m}\left(t\right)}$ represents the mean number of secondary female infections generated per unit time by one infected male in a fully susceptible female population, and

$$\frac{1}{\gamma_{1}^{m}+\mu_{1}^{m}}\cdot \overset{\infty }{\underset{n=0}{\sum }}{\left[\frac{\left(1-p\right)\gamma_{1}^{m}\alpha_{1}^{m}}{\left(\gamma_{1}^{m}+\mu_{1}^{m}\right)\left(\alpha_{1}^{m}+\mu_{1}^{m}\right)}\right]}^{n}=\frac{1}{\gamma_{1}^{m}+\mu_{1}^{m}}\cdot \overset{\infty }{\underset{n=0}{\sum }}\left[\frac{\left(1-p\right)\gamma_{1}^{m}}{\gamma_{1}^{m}+\mu_{1}^{m}}\cdot \frac{\alpha_{1}^{m}}{\alpha_{1}^{m}+\mu_{1}^{m}}\right]$$

represents the duration of the infectious phase in males, including repeated cycles of

$$I_{m}\to C_{m}\to I_{m}.$$

The quantities

$$\frac{\sigma c_{11}^{fm}\beta_{mf}S_{1}^{f}}{N_{1}^{m}\left(t\right)}$$

and

$$\frac{\left(1-p\right)\gamma_{1}^{m}}{\gamma_{1}^{m}+\mu_{1}^{m}}\cdot \frac{1}{\alpha_{1}^{m}+\mu_{1}^{m}}\cdot \overset{\infty }{\underset{n=0}{\sum }}{\left[\frac{\left(1-p\right)\gamma_{1}^{m}\alpha_{1}^{m}}{\left(\gamma_{1}^{m}+\mu_{1}^{m}\right)\left(\alpha_{1}^{m}+\mu_{1}^{m}\right)}\right]}^{n}$$

have analogous interpretations. These two components together constitute the basic reproduction number $R_{0}^{m1}$, that is, the total number of secondary female infections generated by one infected male (whether in the active infectious state *I* or the carrier state *C*) over the course of the entire infectious period. The quantity $R_{0}^{f1}$ has a similar interpretation. Thus, $R_{0}^{11}$ is the basic reproduction number for the entire one-age-group system, representing the threshold quantity for HPV transmission and measuring the average number of secondary infections generated by a typical infected individual introduced into a fully susceptible population.

Case 2 Basic reproduction number for two age groups

Similarly, we can obtain the basic reproduction number (see Appendix B) for two age groups:

$$R_{0}=\rho \left(FV^{-1}\right)=\rho \left(K\right)=\sqrt{\rho \left(K_{mf}K_{fm}\right)}=\sqrt{\frac{b+\sqrt{b^{2}-4c}}{2}},$$

where

$$\begin{array}{ll} b & =x_{9}x_{1}+x_{10}x_{5}+x_{13}x_{2}+x_{14}x_{6},\\ c & =\left(x_{9}x_{1}+x_{10}x_{5}\right)\left(x_{13}x_{2}+x_{14}x_{6}\right)-\left(x_{9}x_{2}+x_{10}x_{6}\right)\left(x_{13}x_{1}+x_{14}x_{5}\right). \end{array}$$

The specific expressions for *x*_1_, *x*_2_, …, *x*_14_ are given in Appendix B.

### 3.3 Local and global stability of the disease-free equilibrium

For the local stability of the DFE, we have the following result.

Theorem 1 *The DFE is locally asymptotically stable when* $R_{0}<1$ *and unstable when* $R_{0}>1$.

**Proof**. Reorder the variables as

$$\begin{array}{ll} x= & \left(I_{1}^{f},\ldots ,I_{n}^{f},C_{1}^{f},\ldots ,C_{n}^{f},I_{1}^{m},\ldots ,I_{n}^{m},C_{1}^{m},\ldots ,C_{n}^{m},S_{1}^{f},\cdots S_{n}^{f},R_{1}^{f},\ldots ,R_{n}^{f},\right)\\ & {\left(S_{1}^{m},\ldots ,S_{n}^{m},R_{1}^{m},\ldots ,R_{n}^{m}\right)}^{T}, \end{array}$$

then the Jacobian matrix at the DFE *E*^0^ is

$$J\left(E_{0}\right)=\left(\begin{array}{llllllll} J_{11} & J_{12} & J_{13} & J_{14} & 0 & 0 & 0 & 0\\ J_{21} & J_{22} & 0 & 0 & 0 & 0 & 0 & 0\\ J_{31} & J_{32} & J_{33} & J_{34} & 0 & 0 & 0 & 0\\ 0 & 0 & J_{43} & J_{44} & 0 & 0 & 0 & 0\\ 0 & 0 & J_{53} & J_{54} & J_{55} & J_{56} & 0 & 0\\ J_{61} & 0 & 0 & 0 & 0 & J_{66} & 0 & 0\\ J_{71} & J_{72} & 0 & 0 & 0 & 0 & J_{77} & J_{78}\\ 0 & 0 & J_{83} & 0 & 0 & 0 & 0 & J_{88} \end{array}\right)=\left(\begin{array}{ll} J_{\text{inf}} & 0\\ J_{\text{mix}\;} & J_{\text{trinf}} \end{array}\right),$$

where

$$\begin{array}{ll} & J_{11}=-V_{II}^{f},\;J_{12}=-V_{IC}^{f},\;J_{13}=F_{13},\;J_{14}=\sigma F_{13},J_{21}=-V_{CI}^{f},\;J_{22}=-V_{CC}^{f},\;J_{31}=J_{71}=F_{31},\\ & J_{32}=J_{72}=\sigma F_{31},\;J_{33}=-V_{II}^{m},\;J_{34}=-V_{IC}^{m},\;J_{43}=-V_{CI}^{m},\;J_{44}=-V_{CC}^{m},\;J_{53}=-F_{13},\\ & J_{54}=-\sigma F_{13},\;J_{55}=X_{1}^{f},\;J_{56}=X_{2}^{f},\;J_{61}=X_{3}^{f},\;J_{66}=X_{4}^{f},\;J_{77}=X_{1}^{m},\;J_{78}=X_{2}^{m},\\ & J_{83}=X_{3}^{m},J_{88}=X_{4}^{m}, \end{array}$$

(3.1) $$\begin{array}{l} X_{1}^{g}={\left(\begin{array}{lllll} -\left(a_{1}^{g}+\mu_{1}^{g}\right) & & & & \\ a_{1}^{g} & -\left(a_{2}^{g}+\mu_{2}^{g}\right) & & & \\ & \ddots & \ddots & & \\ & & a_{n-1}^{g} & -\left(a_{n}^{g}+\mu_{n}^{g}\right) \end{array}\right)}_{n\times n},X_{2}^{g}=\left(\begin{array}{lll} \omega_{1}^{g} & & \\ & \ddots & \\ & & \omega_{n}^{g} \end{array}\right), \end{array}$$

(3.2) $$\begin{array}{l} X_{3}^{g}=\left(\begin{array}{lll} p\gamma_{1}^{g} & & \\ & \ddots & \\ & & p\gamma_{n}^{g} \end{array}\right), \end{array}$$

(3.3) $$\begin{array}{l} X_{4}^{g}=\left(\begin{array}{lllll} -\left(\omega_{1}^{g}+a_{1}^{g}+\mu_{1}^{g}\right) & & & \\ a_{1}^{g} & -\left(\omega_{2}^{g}+a_{2}^{g}+\mu_{2}^{g}\right) & \ddots & \\ & & \ddots & & \ddots \\ & & & a_{n-1}^{g} & -\left(\omega_{n}^{g}+a_{n}^{g}+\mu_{n}^{g}\right) \end{array}\right), \end{array}$$

Therefore, the 8*n* eigenvalues of *J*(*E*_0_) consist of the 4*n* eigenvalues of *J*_inf_ together with the 4*n* eigenvalues of *J*_uninf_.

For the noninfectious subsystem *J*_uninf_, the dynamics are governed only by recruitment, mortality, aging, and waning immunity. This is a standard linear population subsystem, and it naturally approaches a stable population structure:

$$J_{\text{uminf}}=\left(\begin{array}{llll} J_{55} & J_{56} & 0 & 0\\ 0 & J_{66} & 0 & 0\\ 0 & 0 & J_{77} & J_{78}\\ 0 & 0 & 0 & J_{88} \end{array}\right).$$

Clearly, the 4*n* eigenvalues of the matrix *J*_*un*_ _inf_ are

$$-\left(a_{1}^{f}+\mu_{1}^{f}\right),\ldots ,-\left(a_{n}^{f}+\mu_{n}^{f}\right),$$

$$-\left(\omega_{1}^{f}+a_{1}^{f}+\mu_{1}^{f}\right),\ldots ,-\left(\omega_{n}^{f}+a_{n}^{f}+\mu_{n}^{f}\right),-\left(a_{1}^{m}+\mu_{1}^{m}\right),\ldots ,-\left(a_{n}^{m}+\mu_{n}^{m}\right),$$

$$-\left(\omega_{1}^{m}+a_{1}^{m}+\mu_{1}^{m}\right),\ldots ,-\left(\omega_{n}^{m}+a_{n}^{m}+\mu_{n}^{m}\right).$$

All of these eigenvalues have negative real parts.

For the infectious subsystem *J*_inf_, we note that *J*_inf_ = *F* − *V*, where *F* ≥ 0 and *V* is a nonsingular *M*-matrix. Since *V* has positive diagonal entries, nonpositive off-diagonal entries and is strictly diagonally dominant, *V* is a nonsingular M-matrix. We therefore use the following lemma.

Lemma. The following statements are equivalent.

1. *s*(*F* − *V*) < 0 if and only if *ρ*(*FV*^−1^) < 1;
2. *s*(*F* − *V*) > 0 if and only if *ρ*(*FV*^−1^) > 1.

That is,

1. *s*(*J*_inf_) < 0 if and only if $R_{0}<1$;
2. *s*(*J*_inf_) > 0 if and only if $R_{0}>1$.

Hence, when $R_{0}<1$, all eigenvalues of *J*_inf_ have negative real parts, whereas when $R_{0}>1$, at least one eigenvalue of *J*_inf_ has positive real part. Therefore, the DFE is locally asymptotically stable when $R_{0}<1$ and unstable when $R_{0}>1$. This completes the proof of Theorem 1.

For the limiting system, the following result establishes the global stability of the DFE.

Theorem 2 *The DFE is globally asymptotically stable when* $R_{0}<1$.

**Proof**. Since the system with *n* age groups is complicated, we first consider the simple case *n* = 1. Considering the limiting system, that is,

$$N_{1}^{f}=\frac{\Lambda^{f}}{\mu_{1}^{f}},\;N_{1}^{m}=\frac{\Lambda^{m}}{\mu_{1}^{m}},$$

we define the Lyapunov function

$$L=l_{1}I_{1}^{f}+l_{2}C_{1}^{f}+l_{3}I_{1}^{m}+l_{4}C_{1}^{m}$$

with

$$l_{1},\ldots ,l_{4}>0\;\text{are constants,}$$

where *l* = (*l*_1_, *l*_2_, *l*_3_, *l*_4_) satisfies the condition *lFV*^−1^ = *R*_0_*l*,

$$\left(\begin{array}{l} l_{1},l_{2},l_{3},l_{4} \end{array}\right)\left(\begin{array}{llll} 0 & 0 & X_{3} & X_{4}\\ 0 & 0 & 0 & 0\\ X_{1} & X_{2} & 0 & 0\\ 0 & 0 & 0 & 0 \end{array}\right)=R_{0}\left(l_{1},l_{2},l_{3},l_{4}\right),$$

that is,

$$l_{3}X_{1}=R_{0}l_{1},\;l_{3}X_{2}=R_{0}l_{2},\;l_{1}X_{3}=R_{0}l_{3},\;l_{1}X_{4}=R_{0}l_{4},$$

where the expressions for *X*_1_ to *X*_4_ are given in Appendix B.

It is clear that *L* is globally positive definite and radially unbounded on the entire state space. Differentiating *L* along the trajectories of the system gives

$$\begin{array}{ll} \frac{dL}{dt} & =l_{1}\left[\lambda_{1}^{f}S_{1}^{f}+\alpha_{1}^{f}C_{1}^{f}-\left(\gamma_{1}^{f}+\mu_{1}^{f}\right)I_{1}^{f}\right]+l_{2}\left[\left(1-p\right)\gamma_{1}^{f}I_{1}^{f}-\left(\alpha_{1}^{f}+\mu_{1}^{f}\right)C_{1}^{f}\right]\\ & \;+l_{3}\left[\lambda_{1}^{m}S_{1}^{m}+\alpha_{1}^{m}C_{1}^{m}-\left(\gamma_{1}^{m}+\mu_{1}^{m}\right)I_{1}^{m}\right]+l_{4}\left[\left(1-p\right)\gamma_{1}^{m}I_{1}^{m}-\left(\alpha_{1}^{m}+\mu_{1}^{m}\right)C_{1}^{m}\right]. \end{array}$$

Since

$$l_{3}c_{11}^{mf}\frac{\beta_{fm}}{N_{1}^{f0}\left(t\right)}S_{1}^{m0}=R_{0}\left[l_{1}\left(\gamma_{1}^{f}+\mu_{1}^{f}\right)+l_{2}\left(p-1\right)\gamma_{1}^{f}\right],$$

$$l_{3}\sigma c_{11}^{mf}\frac{\beta_{fm}}{N_{1}^{f0}\left(t\right)}S_{1}^{m0}=R_{0}\left[-l_{1}\alpha_{1}^{f}+l_{2}\left(\alpha_{1}^{f}+\mu_{1}^{f}\right)\right],$$

$$l_{4}c_{11}^{fm}\frac{\beta_{mf}}{N_{1}^{m0}}S_{1}^{f0}=R_{0}\left[l_{3}\left(\gamma_{1}^{m}+\mu_{1}^{m}\right)+l_{4}\left(p-1\right)\gamma_{1}^{m}\right],$$

$$l_{4}\sigma c_{11}^{fm}\frac{\beta_{mf}}{N_{1}^{m0}}S_{1}^{f0}=R_{0}\left[-l_{3}\alpha_{1}^{m}+l_{4}\left(\alpha_{1}^{m}+\mu_{1}^{m}\right)\right],$$

the above expressions can be rewritten as

$$\begin{array}{l} -l_{1}\left(\gamma_{1}^{f}+\mu_{1}^{f}\right)+l_{2}\left(1-p\right)\gamma_{1}^{f}=-\frac{l_{3}c_{11}^{mf}\frac{\beta_{fm}}{N_{1}^{f0}}S_{1}^{m0}}{R_{0}},\\ l_{1}\alpha_{1}^{f}-l_{2}\left(\alpha_{1}^{f}+\mu_{1}^{f}\right)=-\frac{l_{3}\sigma c_{11}^{mf}\frac{\beta_{fm}}{N_{1}^{f0}\left(t\right)}S_{1}^{m0}}{R_{0}},\\ -l_{3}\left(\gamma_{1}^{m}+\mu_{1}^{m}\right)+l_{4}\left(1-p\right)\gamma_{1}^{m}=-\frac{l_{1}c_{11}^{fm}\frac{\beta_{mf}}{N_{1}^{m0}}S_{1}^{f0}}{R_{0}},\\ l_{3}\alpha_{1}^{m}-l_{4}\left(\alpha_{1}^{m}+\mu_{1}^{m}\right)=-\frac{l_{1}\sigma c_{11}^{fm}\frac{\beta_{mf}}{N_{1}^{m0}}S_{1}^{f0}}{R_{0}}, \end{array}$$

and therefore

$$\begin{array}{ll} \frac{dL}{dt} & =l_{1}\lambda_{1}^{f}S_{1}^{f}+l_{3}\lambda_{1}^{m}S_{1}^{m}-\frac{1}{R_{0}}\left(l_{3}c_{11}^{mf}\frac{\beta_{fm}}{N_{1}^{f0}}S_{1}^{m0}I_{1}^{f}+l_{3}\sigma c_{11}^{mf}\frac{\beta_{fm}}{N_{1}^{f0}}S_{1}^{m0}C_{1}^{f}\right)\\ & \;\left(+l_{1}c_{11}^{fm}\frac{\beta_{mf}}{N_{1}^{m0}}S_{1}^{f0}I_{1}^{m}+l_{1}\sigma c_{11}^{fm}\frac{\beta_{mf}}{N_{1}^{m0}}S_{1}^{f0}C_{1}^{m}\right). \end{array}$$

Since

$$\lambda_{1}^{f}S_{1}^{f}\leq c_{11}^{fm}\frac{\beta_{mf}\left(I_{1}^{m}+\sigma \cdot C_{1}^{m}\right)N_{1}^{f}}{N_{1}^{m}},\;\lambda_{1}^{m}S_{1}^{m}\leq c_{11}^{mf}\frac{\beta_{fm}\left(I_{1}^{f}+\sigma \cdot C_{1}^{f}\right)N_{1}^{m}}{N_{1}^{f}},$$

it follows that

$$l_{1}\lambda_{1}^{f}S_{1}^{f}\leq l_{1}c_{11}^{fm}\frac{\beta_{mf}\left(I_{1}^{m}+\sigma \cdot C_{1}^{m}\right)N_{1}^{f}}{N_{1}^{m}},\;l_{3}\lambda_{1}^{m}S_{1}^{m}\leq l_{3}c_{11}^{mf}\frac{\beta_{fm}\left(I_{1}^{f}+\sigma \cdot C_{1}^{f}\right)N_{1}^{m}}{N_{1}^{f}},$$

when

$$N\geq \frac{N_{1}^{f0}}{S_{1}^{m0}}S_{1}^{m}+\frac{N_{1}^{m0}}{S_{1}^{f0}}S_{1}^{f},$$

we have

$$\begin{array}{ll} \frac{dL}{dt} & \leq \left(1-\frac{1}{R_{0}}\right)\left(l_{3}c_{11}^{mf}\frac{\beta_{fm}}{N_{1}^{f0}}I_{1}^{f}S_{1}^{m0}+l_{3}\sigma c_{11}^{mf}\frac{\beta_{fm}}{N_{1}^{f0}}C_{1}^{f}S_{1}^{m0}\right)\\ & \;\left(+l_{1}c_{11}^{fm}\frac{\beta_{mf}}{N_{1}^{m0}}I_{1}^{m}S_{1}^{f0}+l_{1}\sigma c_{11}^{fm}\frac{\beta_{mf}}{N_{1}^{m0}}C_{1}^{m}S_{1}^{f0}\right). \end{array}$$

When $R_{0}\leq 1$, we have $\frac{dL}{dt}\leq 0$, with equality only at the DFE. Therefore, by the Krasovskii-LaSalle theorem (Martcheva, 2015), the DFE is globally stable when $R_{0}\leq 1$. Since the DFE is unique and locally asymptotically stable, it follows that the DFE is globally asymptotically stable.

The proof for the case of two age groups is provided in Appendix C. Using the same approach, one can establish global stability for the general case of *n* age groups.

### 3.4 The endemic equilibrium

Theorem 4 *The endemic equilibrium of system* (1) *exists when* $R_{0}>1$.

**Proof**. To determine the endemic equilibrium, we set the right-hand side of system (1) equal to zero:

$$\left\{\begin{array}{l} 0=\Lambda^{g}\delta_{i}+\omega_{i}^{g}R_{i}^{g}-\lambda_{i}^{g}\left(t\right)S_{i}^{g}-\left(a_{i}^{g}+\mu_{i}^{g}\right)S_{i}^{g}+a_{i-1}^{g}S_{i-1}^{g},\\ 0=\lambda_{i}^{g}\left(t\right)S_{i}^{g}+\alpha_{i}^{g}C_{i}^{g}-\left(\gamma_{i}^{g}+a_{i}^{g}+\mu_{i}^{g}\right)I_{i}^{g}+a_{i-1}^{g}I_{i-1}^{g},\\ 0=\left(1-p\right)\gamma_{i}^{g}I_{i}^{g}-\left(\alpha_{i}^{g}+a_{i}^{g}+\mu_{i}^{g}\right)C_{i}^{g}+a_{i-1}^{g}C_{i-1}^{g},\\ 0=p\gamma_{i}^{g}I_{i}^{g}-\left(\omega_{i}^{g}+a_{i}^{g}+\mu_{i}^{g}\right)R_{i}^{g}+a_{i-1}^{g}R_{i-1}^{g}, \end{array}\right)$$

where

$$\lambda_{i}^{g}\left(t\right)=\overset{n}{\underset{j=1}{\sum }}c_{ij}^{gg^{\prime }}\frac{\beta_{g^{\prime }g}\left(I_{j}^{g^{\prime }}\left(t\right)+\sigma \cdot C_{j}^{g^{\prime }}\left(t\right)\right)}{N_{j}^{g^{\prime }}\left(t\right)},\;g,g^{\prime }\in \left\{f,m\right\}.$$

From the second equilibrium equation, we obtain

$$S_{i}^{g}=\frac{\left(\gamma_{i}^{g}+a_{i}^{g}+\mu_{i}^{g}\right)I_{i}^{g}-\alpha_{i}^{g}C_{i}^{g}-a_{i-1}^{g}I_{i-1}^{g}}{\lambda_{i}^{g}\left(t\right)}.$$

From the fourth equilibrium equation, we have

$$R_{i}^{g}=\frac{p\gamma_{i}^{g}I_{i}^{g}+a_{i-1}^{g}R_{i-1}^{g}}{\omega_{i}^{g}+a_{i}^{g}+\mu_{i}^{g}}.$$

Thus, $S_{i}^{g}$ can be expressed as a function of $I_{i}^{g}$ and $C_{i}^{g}$ (*i* = 1, …, *n*, *g* ∈ {*f*, *m*}). Furthermore, $R_{i}^{g}$ can also be regarded as a function of $I_{i}^{g}$ and $C_{i}^{g}$ (*i* = 1, …, *n*, *g* ∈ {*f*, *m*}). In this way, we obtain a closed system involving only the infected compartments. Therefore, in the subsequent analysis, we consider only the equilibrium equations for $I_{i}^{g}$ and $C_{i}^{g}$.

Because of the complexity of the system, it is difficult to solve it directly. We therefore turn to a fixed-point argument.

For convenience, we recall the following theorem (Guo & Lakshmikantham, 2023): let $K\subset R^{n}$ be a cone, and let *A*: *K* → *K* be a compact and continuous operator. Suppose there exist constants 0 < *r* < *R* such that.

1. for all *x* ∈ *K* with ‖*x*‖ = *r*, ‖*A*(*x*)‖ ≥‖*x*‖,
2. for all *x* ∈ *K* with ‖*x*‖ = *R*, ‖*A*(*x*)‖ ≤ ‖*x*‖.

Then *A* has at least one fixed point in the set {*x* ∈ *K*: *r* ≤ ‖*x*‖ ≤ *R*}.

We now use this theorem to show that system (1) has an endemic equilibrium. At equilibrium, we replace $S_{i}^{g}$ in the equilibrium equations for $I_{i}^{g}$ and $C_{i}^{g}$ by $N_{i}^{g}-I_{i}^{g}-C_{i}^{g}-R_{i}^{g}$, where $N^{g*}=\sum_{j=1}^{n}N_{j}^{g*}=\frac{\Lambda^{g}}{\mu^{g}}$ for *g* ∈ {*f*, *m*}. This leads to a nonlinear system in the infected variables. Since the equilibrium equations of system (1) can be written as

$$\frac{dx}{dt}=F\left(x,S\left(x\right)\right)-V\left(x\right)=F\left(x,S\left(x\right)\right)-Vx=0,$$

we rewrite them as the fixed-point equation *x* = *A*(*x*), where

$$x={\left(I_{1}^{f},\ldots ,I_{n}^{f},C_{1}^{f},\ldots ,C_{n}^{f},I_{1}^{m},\ldots ,I_{n}^{m},C_{1}^{m},\ldots ,C_{n}^{m}\right)}^{T},\;A=V^{-1}F,$$

with

$$x\in K=\left\{\left(I_{1}^{f},\ldots ,I_{n}^{f},C_{1}^{f},\ldots ,C_{n}^{f},I_{1}^{m},\ldots ,I_{n}^{m},C_{1}^{m},\ldots ,C_{n}^{m}\right)\in R_{+}^{4n}:\overset{n}{\underset{j=1}{\sum }}I_{j}^{g}+C_{j}^{g}\leq \frac{\Lambda^{g}}{\mu^{g}},\;g\in \left\{f,m\right\}\right\}.$$

Here F and V are the same as those in Section 3.2, and their Jacobians are *F* and *V*, respectively. It is clear that system (∗) has the trivial fixed point

$$\epsilon^{0}=\left(I_{1}^{f0},\ldots ,I_{n}^{f0},C_{1}^{f0},\ldots ,C_{n}^{f0},I_{1}^{m0},\ldots ,I_{n}^{m0},C_{1}^{m0},\ldots ,C_{n}^{m0}\right)=\left(0,0,\ldots ,0\right).$$

We compute the Jacobian of *A* at *ɛ*^0^:

$$A^{\prime }\left(\epsilon^{0}\right)=V^{-1}F,$$

$$\rho \left(A^{\prime }\left(\epsilon^{0}\right)\right)=\rho \left(V^{-1}F\right)=\rho \left(FV^{-1}\right)=R_{0}.$$

Since *A* is compact, it is completely continuous. In addition, *V* is a nonsingular *M*-matrix, so its inverse *V*^−1^ is nonnegative. Because of the full connectivity of the contact network, the new infection matrix *F* is irreducible. It follows that *A*′(*ɛ*^0^) is nonnegative and irreducible. By the Perron-Frobenius theorem, there exists a positive eigenvector *v* > 0 corresponding to $R_{0}$, that is, $A^{\prime }\left(\epsilon^{0}\right)v=R_{0}v$. For any sufficiently small *ɛ* > 0, we have

$$A\left(\epsilon v\right)=A^{\prime }\left(\epsilon^{0}\right)\left(\epsilon v\right)+\left(\begin{array}{l} o\left(\epsilon \right)\\ \vdots \\ o\left(\epsilon \right) \end{array}\right)=\epsilon R_{0}v+\left(\begin{array}{l} o\left(\epsilon \right)\\ \vdots \\ o\left(\epsilon \right) \end{array}\right).$$

When $R_{0}>1$, it follows that ‖*A*(*ɛv*)‖ > ‖*ɛv*‖.

We also use the following lemma (N’Guérékata & O’Regan, 2022): there exists a sufficiently large constant *M* > 0 such that for any solution (*x*, *c*) satisfying *x* = *cA*(*x*), where *x* ∈ *K* ∖{0} and *c* ≥ 1, one has ‖*x*‖ ≤ *M*.

Next, we use a contradiction argument to show that the operator *A* is contractive at infinity. Let

$$\epsilon^{\infty }=\left(I_{1}^{f\infty },\ldots ,I_{n}^{f\infty },C_{1}^{f\infty },\ldots ,C_{n}^{f\infty },I_{1}^{m\infty },\ldots ,I_{n}^{m\infty },C_{1}^{m\infty },\ldots ,C_{n}^{m\infty }\right).$$

Suppose there exists a sequence of solutions $\left\{\left(x_{k},c_{k}\right)\right\}$, where *x*_*k*_ ∈ *K* ∖{0} and *c*_*k*_ ≥ 1, satisfying

$$x_{k}=c_{k}A\left(x_{k}\right),\;\|x_{k}\|\to \infty \;\text{as}\;k\to \infty .$$

Substituting the definition of *A* into this equation gives

$$x_{k}=c_{k}V^{-1}F\left(x_{k}\right).$$

Multiplying both sides by *V*, we obtain

$$Vx_{k}=c_{k}F\left(x_{k}\right).$$

Since *x*_*k*_ belongs to the positive cone *K*, all of its components $\left(I_{i}^{g},C_{i}^{g}\right)$ are nonnegative. Moreover, $S_{i}^{g}$ and $R_{i}^{g}$ are also nonnegative. Therefore, for any component of *x*_*k*_, such as $C_{i}^{g}$, we have

$$C_{i}^{g}\left(x_{k}\right)\leq N_{i}^{g}.$$

Similarly, all components of *x*_*k*_ are bounded above by the corresponding total populations $N_{i}^{g}$. This implies that the norm ‖*x*_*k*_‖ is also bounded. For example, we obtain the rough estimate

$$\|x_{k}\|\leq \underset{g\in \left\{f,m\right\}}{\sum }\overset{n}{\underset{j=1}{\sum }}N_{j}^{g}=N_{\text{total}},$$

where *N*_total_ denotes the total population of the system, which is independent of *k*. This contradicts the assumption that ‖*x*_*k*_‖ → *∞*. Therefore, there exists a sufficiently large radius *M*′ such that ‖*A*(*x*)‖ < ‖*x*‖ whenever ‖*x*‖ > *M*′. Hence, *A* has at least one fixed point in *K*, and therefore system (1) has at least one equilibrium. Since *x*∗ ≠ 0 and *x*∗ ≥ 0, there exists an endemic equilibrium. This completes the proof.

Because of the complexity of the model, it is difficult to study analytically the uniqueness and stability of the endemic equilibrium. According to the numerical simulations under different initial conditions presented later (Fig. 2), the endemic equilibrium appears to be unique and stable.

**Fig. 2:**
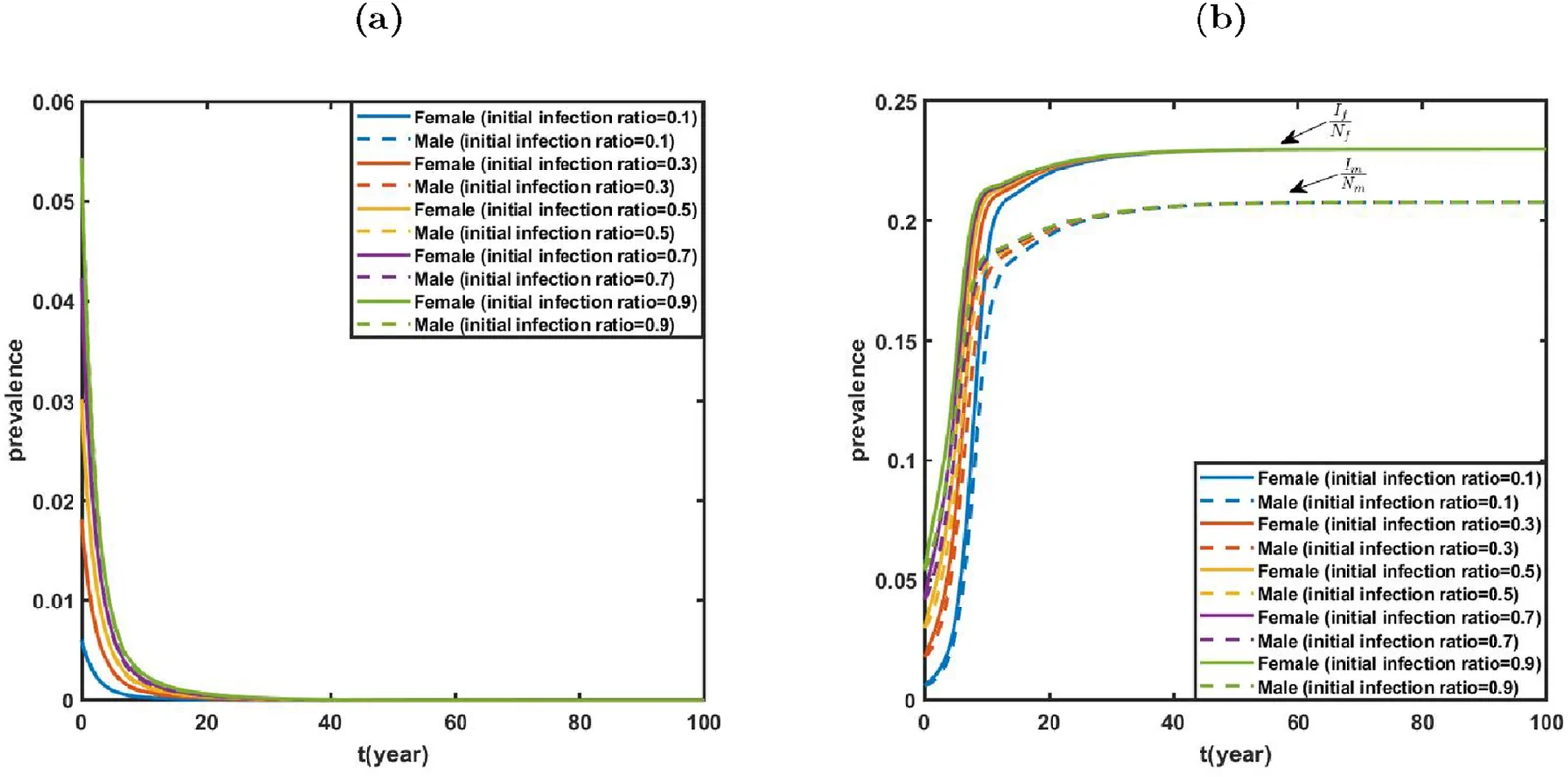
Predicted prevalence dynamics of model (1) with *n* = 5. The threshold value $R_{0}$ is 0.6097 in panel (a) and 2.5263 in panel (b). Panel (a) shows that the disease dies out, whereas panel (b) shows that solutions corresponding to different initial conditions converge to the same endemic equilibrium. The parameter values are *β*_*mf*_ = 0.15 and *β*_*fm*_ = 0.2 in panel (a), and *β*_*mf*_ = 0.6082 and *β*_*fm*_ = 0.6239 in panel (b). All other parameters are the same as those listed in Table (3).

### **3.5** **Analytical results, scope, and limitations of the general*n*-age-group model**

For one-age-group or two-age-group model, the disease-free equilibrium and the basic reproduction number can be obtained in explicit forms. In addition, the disease-free equilibrium is shown to be both locally asymptotically stable and globally asymptotically stable under the corresponding threshold condition.

For the general *n*-age-group model, an explicit expression for the disease-free equilibrium is derived. The basic reproduction number is represented in the standard next-generation matrix form:

$$R_{0}=\rho \left(FV^{-1}\right),$$

where *ρ*(⋅) denotes the spectral radius, F and V have the same meaning as above. The disease-free equilibrium of the general model is proved to be locally asymptotically stable and globally asymptotically stable when $R_{0}<1$. The global stability result is obtained by extending the Lyapunov-function argument established for the one-age-group and two-age-group systems to the general *n*-dimensional setting. Thus, the result of *n*-age-group is a genuine generalization under assumptions, rather than an inference based solely on the lower-dimensional cases. For the endemic equilibrium, an implicit characterization is obtained and its existence is established. Although the stability and uniqueness of the endemic equilibrium are not established analytically for arbitrary *n*, the numerical investigations presented in the Numerical investigations Section provide numerical evidence supporting the stability and uniqueness of the endemic equilibrium under the parameter settings considered.

## 4 Numerical investigations

### 4.1 Parameter setting and data fitting

Most parameter values are taken from previous modeling studies and epidemiological literature. We divide the population into five age groups: 15-24 years, 25-34 years, 35-44 years, 45-54 years, and >54 years. Each of the first four age groups spans 10 years. In age-structured models, especially age-classified models in infectious disease dynamics or population dynamics, the last group is usually treated as the terminal age group, with no further age-related outflow. Therefore, we set

$$a_{1}=\frac{1}{10},a_{2}=\frac{1}{10},a_{3}=\frac{1}{10},a_{4}=\frac{1}{10}\;\;\text{and}\;\;a_{5}=0.$$

Based on data from the National Bureau of Statistics, the population mortality rate in 2011 was 7.14%. Given the relatively higher mortality rate observed in the older age group, we assume a mortality rate of 0.12 for the final age group. The mortality rates for the first four age groups are assumed to be identical, and the mortality for females is assumed to be moderately lower than that for males. That is

$$\mu_{1}^{m}=\mu_{2}^{m}=\mu_{3}^{m}=\mu_{4}^{m}=0.0714,$$

$$\mu_{1}^{f}=\mu_{2}^{f}=\mu_{3}^{f}=\mu_{4}^{f}=0.9\times 0.0714,$$

$$\mu_{5}^{m}=0.12,\;\mu_{5}^{f}=0.9\times 0.12.$$

According to Johnson et al. (2001) (Johnson et al., 2001), together with the observation that individuals primarily have sexual contact with opposite-sex partners in the same age group, with a smaller proportion of contacts occurring with adjacent age groups and very limited contact across more distant age groups (Antfolk, 2017), we define the following two contact matrices (their visualization is shown in Fig. 3):

$$C^{mf}=\left(\begin{array}{lllll} c_{11}^{mf} & c_{12}^{mf} & c_{13}^{mf} & c_{14}^{mf} & c_{15}^{mf}\\ c_{21}^{mf} & c_{22}^{mf} & c_{23}^{mf} & c_{24}^{mf} & c_{25}^{mf}\\ c_{31}^{mf} & c_{32}^{mf} & c_{33}^{mf} & c_{34}^{mf} & c_{35}^{mf}\\ c_{41}^{mf} & c_{42}^{mf} & c_{43}^{mf} & c_{44}^{mf} & c_{45}^{mf}\\ c_{51}^{mf} & c_{52}^{mf} & c_{53}^{mf} & c_{54}^{mf} & c_{55}^{mf} \end{array}\right)=\left(\begin{array}{lllll} 3 & 0.1 & 0.01 & 0.01 & 0.01\\ 0.1 & 2 & 0.1 & 0.01 & 0.01\\ 0.01 & 0.1 & 1 & 0.1 & 0.01\\ 0.01 & 0.01 & 0.1 & 1 & 0.1\\ 0.01 & 0.01 & 0.01 & 0.05 & 0.7 \end{array}\right)$$

$$C^{fm}=\left(\begin{array}{lllll} c_{11}^{fm} & c_{12}^{fm} & c_{13}^{fm} & c_{14}^{fm} & c_{15}^{fm}\\ c_{21}^{fm} & c_{22}^{fm} & c_{23}^{fm} & c_{24}^{fm} & c_{25}^{fm}\\ c_{31}^{fm} & c_{32}^{fm} & c_{33}^{fm} & c_{34}^{fm} & c_{35}^{fm}\\ c_{41}^{fm} & c_{42}^{fm} & c_{43}^{fm} & c_{44}^{fm} & c_{45}^{fm}\\ c_{51}^{fm} & c_{52}^{fm} & c_{53}^{fm} & c_{54}^{fm} & c_{55}^{fm} \end{array}\right)=\left(\begin{array}{lllll} 2 & 0.1 & 0.01 & 0.01 & 0.01\\ 0.1 & 1 & 0.1 & 0.01 & 0.01\\ 0.01 & 0.1 & 1 & 0.1 & 0.01\\ 0.01 & 0.01 & 0.1 & 1 & 0.1\\ 0.01 & 0.01 & 0.01 & 0.05 & 0.5 \end{array}\right)$$

**Fig. 3:**
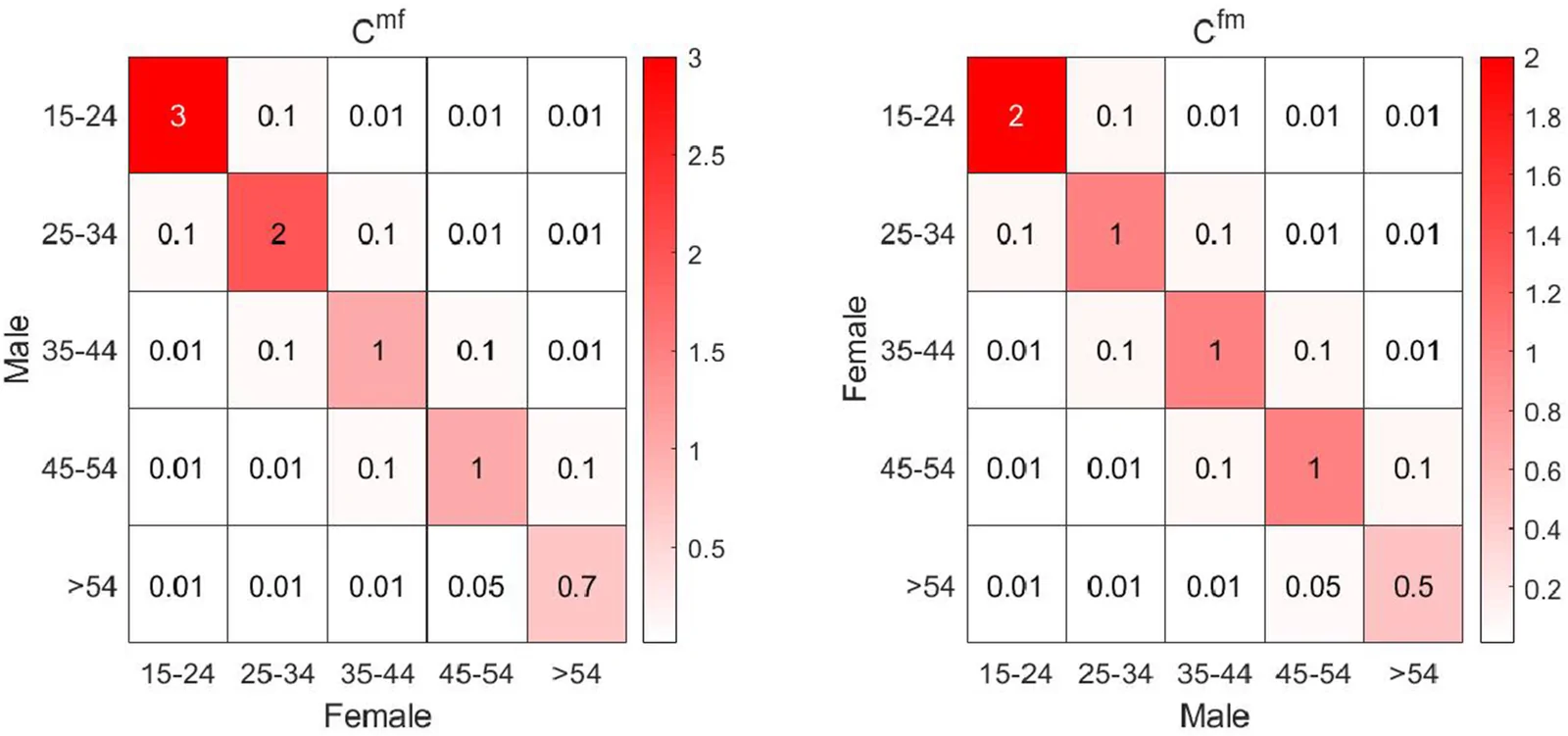
Heatmap of the male-female contact matrix. Sexual contacts are concentrated mainly between opposite-sex individuals of similar age, whereas contacts between individuals with large age differences are relatively rare.

The values of the remaining parameters are taken from previous studies, except for *β*_*mf*_ and *β*_*fm*_; detailed information is provided in Table 3. Furthermore, to study the disease dynamics themselves rather than demographic changes, we assume a constant total population. That is, the recruitment rate is set equal to the mortality rate. Since the numbers of young males and females are not substantially different, we assume

$$\Lambda^{f}=\Lambda^{m}=\frac{\sum_{i=1}^{5}\left[\mu_{i}^{f}\left(S_{i}^{f}+I_{i}^{f}+C_{i}^{f}+R_{i}^{f}\right)+\mu_{i}^{m}\left(S_{i}^{m}+I_{i}^{m}+C_{i}^{m}+R_{i}^{m}\right)\right]}{2}.$$

The assumption of the recruitment rate is equal simplifies the demographic structure of the model while allowing the analysis to focus on the transmission dynamics. This assumption is consistent with similar settings adopted in previous mathematical models(Mukandavire et al. (2007)).

**Table 3:** Fixed parameters in model (1).

Symbol	Baseline	Unit	Source
*ω*_*f*_	0.5	year^−1^	[Assumed]
*ω*_*m*_	0.65	year^−1^	[Assumed]
*a*_1_	1/10	year^−1^	See text
*a*_2_	1/10	year^−1^	See text
*a*_3_	1/10	year^−1^	See text
*a*_4_	1/10	year^−1^	See text
*a*_5_	0	year^−1^	See text
$\mu_{1}^{f}$	0.06426	year^−1^	See text
$\mu_{2}^{f}$	0.06426	year^−1^	See text
$\mu_{3}^{f}$	0.06426	year^−1^	See text
$\mu_{4}^{f}$	0.06426	year^−1^	See text
$\mu_{5}^{f}$	0.108	year^−1^	See text
$\mu_{1}^{m}$	0.0714	year^−1^	See text
$\mu_{2}^{m}$	0.0714	year^−1^	See text
$\mu_{3}^{m}$	0.0714	year^−1^	See text
$\mu_{4}^{m}$	0.0714	year^−1^	See text
$\mu_{5}^{m}$	0.12	year^−1^	See text
$\alpha_{1}^{f}$	0.04	year^−1^	Varied
$\alpha_{2}^{f}$	0.05	year^−1^	Varied
$\alpha_{3}^{f}$	0.06	year^−1^	Varied
$\alpha_{4}^{f}$	0.07	year^−1^	Varied
$\alpha_{5}^{f}$	0.08	year^−1^	Varied
$\alpha_{1}^{m}$	0.06	year^−1^	Varied
$\alpha_{2}^{m}$	0.07	year^−1^	Varied
$\alpha_{3}^{m}$	0.08	year^−1^	Varied
$\alpha_{4}^{m}$	0.09	year^−1^	Varied
$\alpha_{5}^{m}$	0.10	year^−1^	Varied
$\gamma_{1}^{f}$	0.7037	year^−1^	Yulin et al., 2023Yulin et al. (2023)
$\gamma_{2}^{f}$	0.7714	year^−1^	Yulin et al., 2023Yulin et al. (2023)
$\gamma_{3}^{f}$	0.7593	year^−1^	Yulin et al., 2023Yulin et al. (2023)
$\gamma_{4}^{f}$	0.6195	year^−1^	Yulin et al., 2023Yulin et al. (2023)
$\gamma_{5}^{f}$	0.3226	year^−1^	Yulin et al., 2023Yulin et al. (2023)
$\gamma_{1}^{m}$	0.783	year^−1^	Ingles et al., 2015Ingles et al. (2015)
$\gamma_{2}^{m}$	0.6583	year^−1^	Ingles et al., 2015Ingles et al. (2015)
$\gamma_{3}^{m}$	0.583	year^−1^	Ingles et al., 2015Ingles et al. (2015)
$\gamma_{4}^{m}$	0.55	year^−1^	Ingles et al., 2015Ingles et al. (2015)
$\gamma_{5}^{m}$	0.533	year^−1^	Ingles et al., 2015Ingles et al. (2015)
*p*	0.9	unitless	Jiménez-Wences et al., 2014Jiménez-Wences et al. (2014)
*σ*	0.5	unitless	[Assumed]

Finally, as an example, following Qian et al. (2017) (Qian et al., 2017), we use data from gynecological outpatients in Hangzhou in 2011 as initial values and assume that the number of infected males is slightly higher than that of infected females. The initial values are given by

$$I_{1}^{f0}=5,\;I_{2}^{f0}=207,\;I_{3}^{f0}=332,\;I_{4}^{f0}=404,\;I_{5}^{f0}=185,$$

$$S_{1}^{f0}=101,\;S_{2}^{f0}=839,\;S_{3}^{f0}=1120,\;S_{4}^{f0}=1222,\;S_{5}^{f0}=586,$$

$$I_{1}^{m0}=1.06\times I_{1}^{f0},\;I_{2}^{m0}=1.06\times I_{2}^{f0},\;I_{3}^{m0}=1.06\times I_{3}^{f0},\;I_{4}^{m0}=1.06\times I_{4}^{f0},\;I_{5}^{m0}=1.06\times I_{5}^{f0},$$

$$S_{1}^{m0}=1.05\times N_{1}^{f0}-I_{1}^{m0},\;S_{2}^{m0}=1.05\times N_{2}^{f0}-I_{2}^{m0},\;S_{3}^{m0}=1.05\times N_{3}^{f0}-I_{3}^{m0},$$

$$S_{4}^{m0}=1.05\times N_{4}^{f0}-I_{4}^{m0},\;S_{5}^{m0}=1.05\times N_{5}^{f0}-I_{5}^{m0}.$$

For convenience, all remaining compartments are set to zero.

Fig. 4 shows the time-series least-squares fit of model (1), comparing the model output for female prevalence with the observed data for different age groups in Hangzhou from 2011 to 2015. The goodness of fit is *R*^2^ = 0.9855. In general, the closer *R*^2^ is to 1, the better the model fits the data, whereas values closer to 0 indicate poorer agreement. Thus, the value obtained here suggests that the model provides a good fit to the data. We define SSE as the sum of squared errors and SST as the total sum of squares. The formula for *R*^2^ is

$$R^{2}=1-\frac{SSE}{SST},$$

$$SSE=\overset{n}{\underset{i=1}{\sum }}{\left(y_{i}-{\hat{y}}_{i}\right)}^{2},\;SST=\overset{n}{\underset{i=1}{\sum }}{\left(y_{i}-\bar{y}\right)}^{2},$$

where *y*_*i*_ denotes the observed value, ${\hat{y}}_{i}$ the predicted value, and $\bar{y}$ the mean of the observed values. The fitted parameter values of model (1) are *β*_*mf*_ = 0.42755 and *β*_*fm*_ = 0.681048. Substituting all parameter values into the expression for $R_{0}$, we obtain the basic reproduction number for the five-age-group system as $R_{0}=2.177$.

**Fig. 4:**
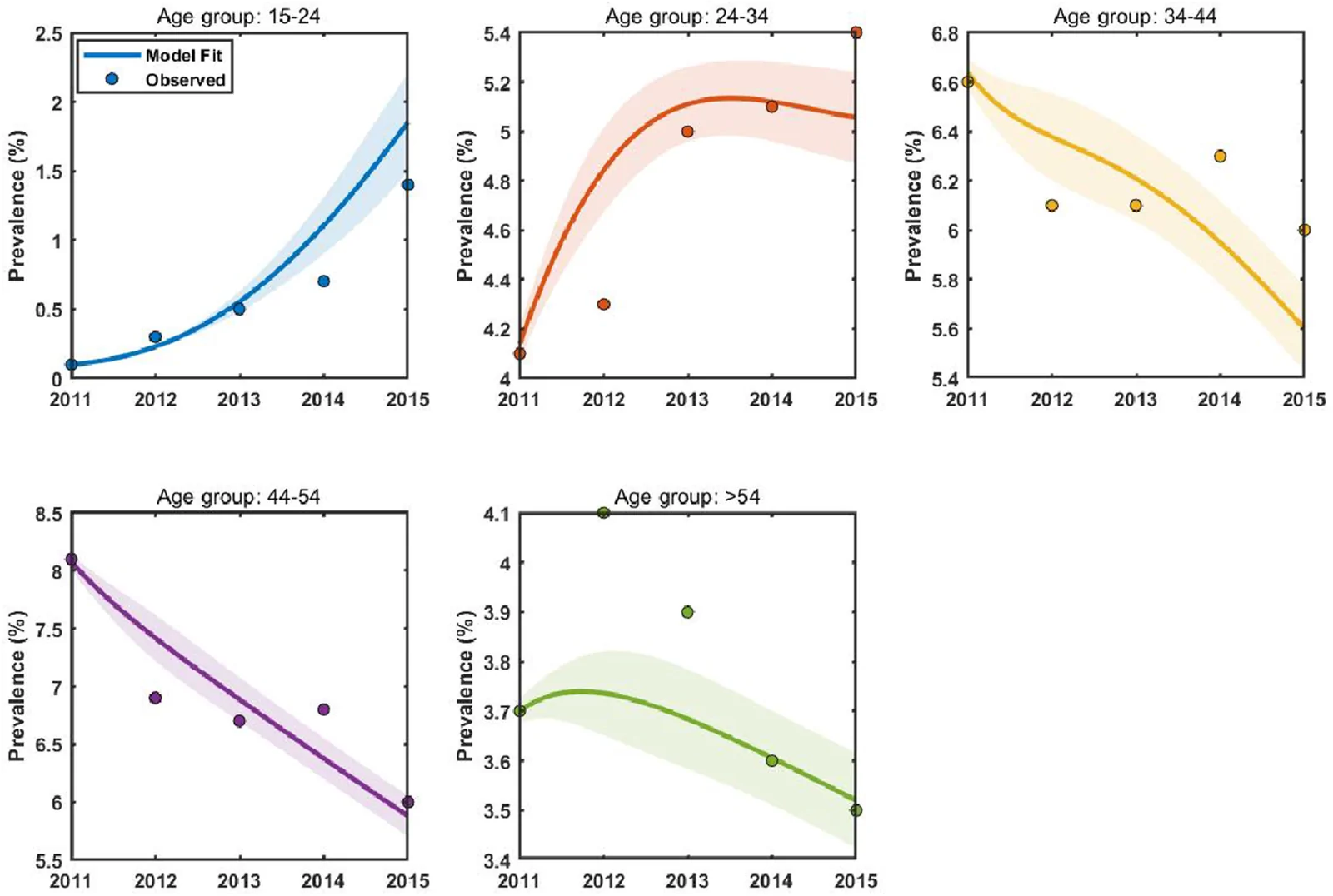
Least-squares fitting results for HPV prevalence in each female age group (*R*^2^ = 0.9855). Model (1) with *n* = 5 is fitted to observed female prevalence data from Hangzhou during 2011-2015.

### 4.2 Sensitivity analysis

Because viral reactivation may play an important role in the transmission of sexually transmitted infections, we are particularly interested in the influence of $\alpha_{i}^{g}$ on HPV transmission dynamics. In this section, we use the PRCC (partial rank correlation coefficient) method to evaluate the effects of model parameters on the dynamics of model (1) (see Fig. 5). Here, *n* = 5. We focus on the ten viral reactivation rates $\left(\alpha_{1}^{f},\;\alpha_{2}^{f},\;\alpha_{3}^{f},\;\alpha_{4}^{f},\;\alpha_{5}^{f},\alpha_{1}^{m},\;\alpha_{2}^{m},\;\alpha_{3}^{m},\;\alpha_{4}^{m},\;\alpha_{5}^{m}\right)$, since these parameters directly govern the core transition from the latent carrier state (*C*) to active infection (*I*) in the model and are therefore central to understanding the long-term transmission dynamics of HPV. By fixing all other parameters and varying only the reactivation rates, we simplify the analysis while still accurately assessing their independent roles in shaping HPV epidemic trends. This allows us to clearly examine how heterogeneity in viral reactivation contributes to the persistence of HPV transmission.

**Fig. 5:**
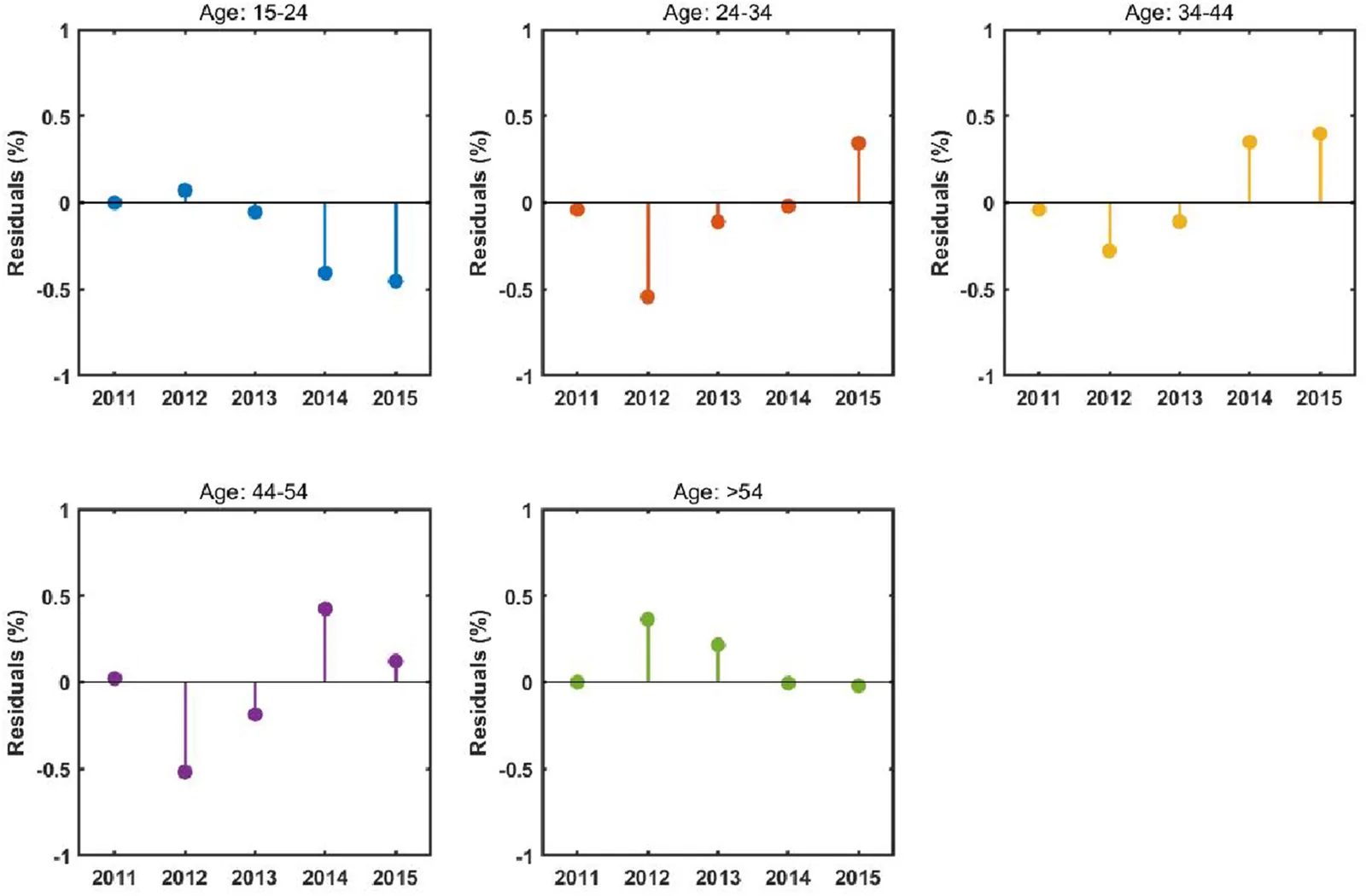
Residuals (different between the observed data and the model fit) in function of time. The random behavior observed in the residuals confirmed the validity of the fit.

PRCC provides a global sensitivity analysis tool for nonlinear but monotone relationships between inputs and outputs (Marino et al., 2008). From Section 3.3, we know that $R_{0}<1$ is required for disease elimination. In addition, we are particularly concerned with the influence of viral reactivation rates on $R_{0}$ and on prevalence at the endemic equilibrium. From Fig. 6a–d, we observe that the prevalence approaches its endemic state approximately 150 years after 2011. Therefore, in the sensitivity analysis, the inputs are the viral reactivation rates for males and females across the five age groups, and the outputs are $R_{0}$, the prevalence in each sex, and the prevalence in the total population at year 150. Among the inputs, we include a special parameter called *dummy*. This parameter does not appear in the model and therefore should not affect the outputs. Ideally, its PRCC value should be zero. In practice, however, numerical artifacts may arise, for example because of aliasing or interference effects. We therefore introduce the dummy parameter to quantify such artifacts. Parameters with sensitivity indices less than or equal to that of the dummy parameter should be regarded as not significantly different from zero.

**Fig. 6:**
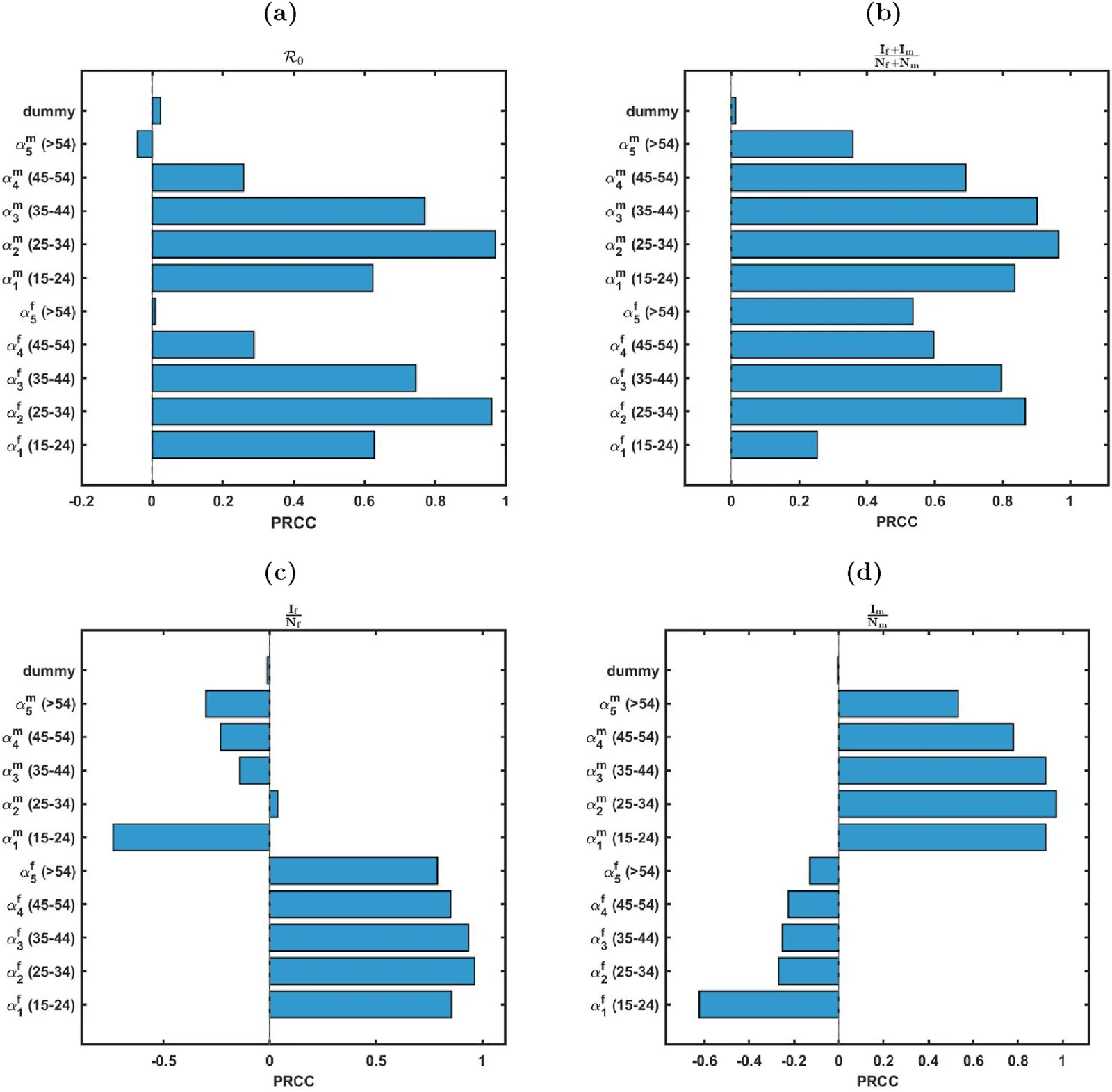
Sensitivity analysis based on PRCC. The baseline values of $\alpha_{1}^{f},\alpha_{2}^{f},\alpha_{3}^{f},\alpha_{4}^{f},\alpha_{5}^{f},\alpha_{1}^{m},\alpha_{2}^{m},\alpha_{3}^{m},\alpha_{4}^{m}$, and $\alpha_{5}^{m}$ are set to 0.04, 0.05, 0.06, 0.07, 0.08, 0.06, 0.07, 0.08, 0.09, and 0.1, respectively. The corresponding parameter ranges are [0.02, 0.06], [0.025, 0.075], [0.03, 0.09], [0.035, 0.105], [0.04, 0.12], [0.03, 0.09], [0.035, 0.105], [0.04, 0.12], [0.045, 0.135], and [0.05, 0.15], respectively.

Korostil et al. found that the reactivation rate of HPV increases with age (Korostil & Regan, 2014). Accordingly, in all sensitivity analyses we choose 0.04, 0.05, 0.06, 0.07, 0.08, 0.06, 0.07, 0.08, 0.09, and 0.1 as the baseline values for $\alpha_{1}^{f}$, $\alpha_{2}^{f}$, $\alpha_{3}^{f}$, $\alpha_{4}^{f}$, $\alpha_{5}^{f}$, $\alpha_{1}^{m}$, $\alpha_{2}^{m}$, $\alpha_{3}^{m}$, $\alpha_{4}^{m}$, and $\alpha_{5}^{m}$, respectively, and define the perturbation range as ± 50% of each baseline value. The sample size is 10000 for all parameters. Parameters with larger PRCC indices (absolute values greater than 0.5) are considered to have stronger effects on the outputs (Taylor, 1990). The sign of the PRCC value indicates whether the parameter is negatively or positively correlated with the corresponding output.

From Fig. 6a–d, the PRCC results for the basic reproduction number show that the four most influential parameters are the transition rates from $C_{2}^{f}$ to $I_{2}^{f}$ $\left(\alpha_{2}^{f}\right)$, from $C_{3}^{f}$ to $I_{3}^{f}$ $\left(\alpha_{3}^{f}\right)$, from $C_{2}^{m}$ to $I_{2}^{m}$ $\left(\alpha_{2}^{m}\right)$, and from $C_{3}^{m}$ to $I_{3}^{m}$ $\left(\alpha_{3}^{m}\right)$. At the endemic equilibrium, the PRCC results for the total prevalence $\frac{I_{f}+I_{m}}{N}$ show that the reactivation rates of all ten viruses have an impact on the overall prevalence. The PRCC results for $\frac{I_{f}}{N_{f}}$ show that the six most influential parameters are the transition rates from $C_{1}^{f}$ to $I_{1}^{f}$ $\left(\alpha_{1}^{f}\right)$, from $C_{2}^{f}$ to $I_{2}^{f}$ $\left(\alpha_{2}^{f}\right)$, from $C_{3}^{f}$ to $I_{3}^{f}$ $\left(\alpha_{3}^{f}\right)$, from $C_{4}^{f}$ to $I_{4}^{f}$ $\left(\alpha_{4}^{f}\right)$, from $C_{5}^{f}$ to $I_{5}^{f}$ $\left(\alpha_{5}^{f}\right)$, and from $C_{1}^{m}$ to $I_{1}^{m}$ $\left(\alpha_{1}^{m}\right)$, and the PRCC results for $\frac{I_{m}}{N_{m}}$ show that the six most influential parameters are the transition rates from $C_{1}^{m}$ to $I_{1}^{m}$ $\left(\alpha_{1}^{m}\right)$, from $C_{2}^{m}$ to $I_{2}^{m}$ $\left(\alpha_{2}^{m}\right)$, from $C_{3}^{m}$ to $I_{3}^{m}$ $\left(\alpha_{3}^{m}\right)$, from $C_{4}^{m}$ to $I_{4}^{m}$ $\left(\alpha_{4}^{m}\right)$, from $C_{5}^{m}$ to $I_{5}^{m}$ $\left(\alpha_{5}^{m}\right)$, and from $C_{1}^{f}$ to $I_{1}^{f}$ $\left(\alpha_{1}^{f}\right)$.

From Fig. 6a and Table 4, we see that viral reactivation rates in the young population (25-34 years) have the greatest influence on $R_{0}$. This suggests that HPV screening and vaccination should be prioritized among 25-34 age group. In particular, male vaccination may have an important effect on reducing transmission. Individuals with high reactivation rates are less likely to remain in a long-term asymptomatic carrier state, which may reduce the long-term infection burden of the epidemic. From Fig. 6b, we observe a positive correlation between the viral reactivation rate and prevalence in the total population for people in all age groups. More generally, viral reactivation rates are positively correlated with total prevalence across all sex and age groups.

**Table 4:** PRCC values for $R_{0}$ and for the prevalence in females, males, and the total population. An asterisk (∗) indicates statistical significance.

Input	PRCC$\left(R_{0}\right)$	PRCC$\left(\frac{I_{f}+I_{m}}{N}\right)$	PRCC$\left(\frac{I_{f}}{N_{f}}\right)$	PRCC$\left(\frac{I_{m}}{N_{m}}\right)$
dummy	0.0237	0.0120	−0.0117	−0.0027
$\alpha_{1}^{f}$	0.6272∗	0.2534∗	0.8546∗	−0.6247∗
$\alpha_{2}^{f}$	0.9595∗	0.8667∗	0.9611∗	−0.2683∗
$\alpha_{3}^{f}$	0.7459∗	0.7960∗	0.9359∗	−0.2514∗
$\alpha_{4}^{f}$	0.2880∗	0.5982∗	0.8502∗	−0.2249∗
$\alpha_{5}^{f}$	0.0086	0.5364∗	0.7898∗	−0.1281∗
$\alpha_{1}^{m}$	0.6228∗	0.8354∗	−0.7358∗	0.9249∗
$\alpha_{2}^{m}$	0.9698∗	0.9654∗	0.0366	0.9720∗
$\alpha_{3}^{m}$	0.7693∗	0.9023∗	−0.1408∗	0.9251∗
$\alpha_{4}^{m}$	0.2579∗	0.6923∗	−0.2312∗	0.7793∗
$\alpha_{5}^{m}$	−0.0410	0.3579∗	−0.3012∗	0.5346∗

From Fig. 5c and d, the viral reactivation rate in each female age group is positively correlated with female prevalence, whereas the viral reactivation rate in each male age group is negatively correlated with female prevalence. A similar conclusion holds for male prevalence. This negative correlation implies that an excessively low viral reactivation rate is not always beneficial for controlling HPV prevalence in the opposite sex population. Walling and Marinelli also reported similar findings, namely that an excessively low viral reactivation rate is not always favorable for reducing disease prevalence (Walling, Seago, & D, 2024; Marinelli & van Lier, 2016). These results further suggest that prevention and control strategies targeting only one sex may have limited effectiveness, and that effective intervention programs should include both males and females.

### 4.3 Simulation results and epidemiological implications

Under the five-age-group, two-sex population structure considered here, the basic reproduction number is calculated as $R_{0}=2.177$, indicating that HPV can persist in the population.

Fig. 7a and b compare two simulation scenarios and reveal the important regulatory role of carrier infectivity in determining the epidemiological characteristics of HPV transmission. Compared with the case of lower carrier infectivity (*σ* = 0.5), both the number of infectious individuals and the number of carriers increase in all age groups and in both sexes when carrier infectivity is higher (*σ* = 1.5). In particular, when carriers are more infectious than actively infected individuals (*σ* = 1.5), the infection burden and carrier burden increase across all age groups for both females and males. This result suggests that high carrier infectivity can amplify the long-term transmission potential of HPV. In contrast, when carrier infectivity is lower than that of actively infected individuals (*σ* = 0.5), the overall infection burden is reduced. Nevertheless, regardless of the magnitude of carrier infectivity, the qualitative age-distribution pattern remains broadly similar in the two sexes.

**Fig. 7:**
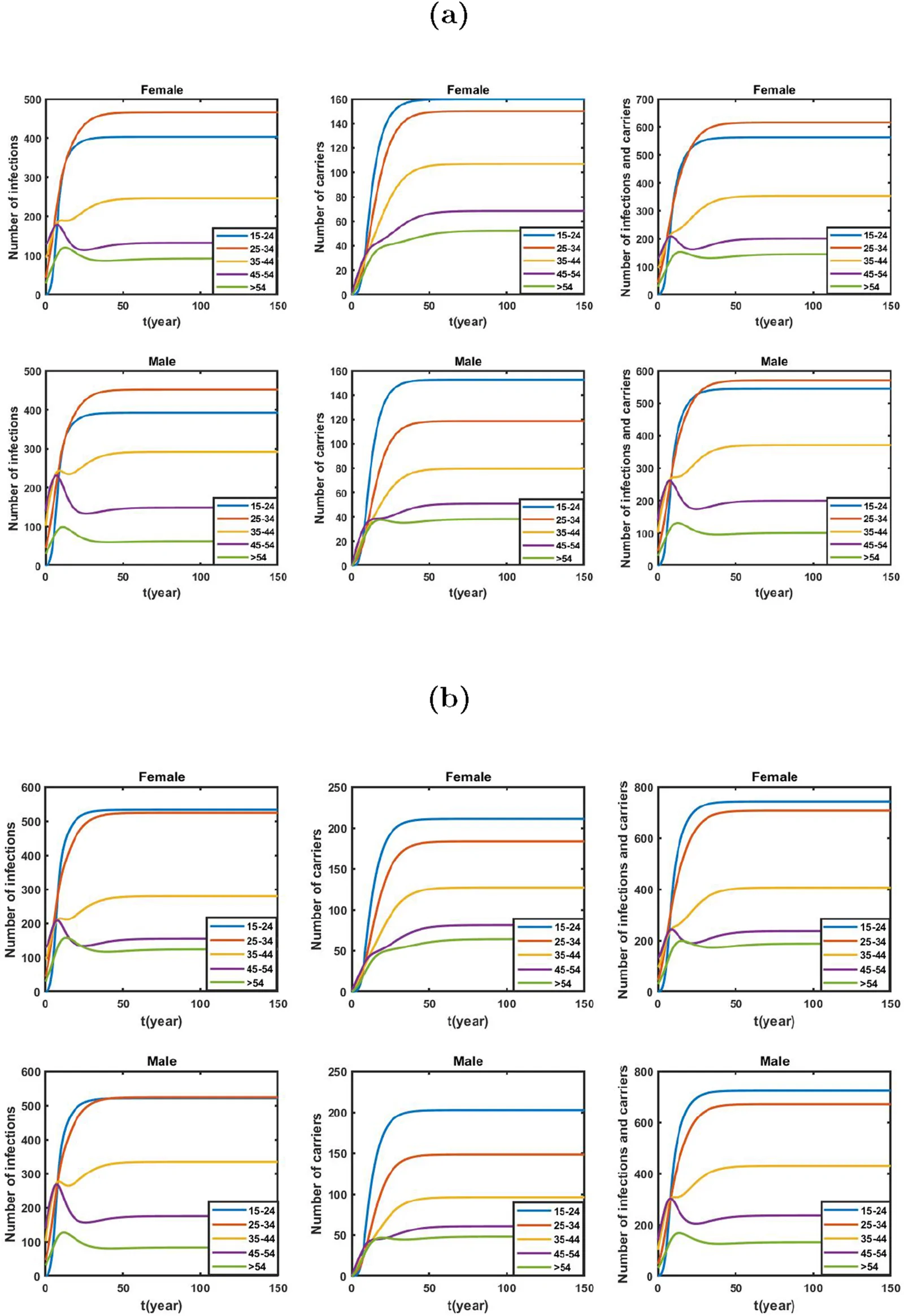
Panels **(a)** and **(b)** show the temporal trends in the numbers of infectious individuals, carriers, and their total across age groups and by sex. In panel **(a)**, $R_{0}=2.177$ and *σ* = 0.5, so the infectivity of virus carriers is lower than that of infectious individuals. In panel **(b)**, $R_{0}=2.7128$ and *σ* = 1.5, so the infectivity of virus carriers is higher than that of infectious individuals.

Since the HPV vaccine was first introduced in China in 2016, we examine the status of HPV infection six years after 2011. From the perspective of viral reservoir distribution (Fig. 8a), the number of carriers among males and females in each age group is similar, suggesting that we need to simultaneously consider and control the viral reactivation rates in both sexes. The results for infected individuals show that infection peaks occur in the 15-24 and 45-54 age groups, which is consistent with the bimodal pattern of HPV infection reported in many previous studies (Campos et al., 2021; Schiffman et al., 2016). Fig. 7, Fig. 8a show that the youngest age group (15-24 years) accounts for the largest proportion of infected individuals (*I*) and thus serves as a major driver of transmission. As age increases, the number of infections in both sexes generally declines, but rises again in the 45-54 age group, a pattern that may be related to viral latency and reactivation. In contrast, females and males in the middle-aged groups (25-54 years) have a higher proportion of carriers. We also consider two extreme scenarios. In Scenario 1, where there is no viral clearance at all, the number of both latent individuals and infected individuals is increasing rapidly, as shown in Fig. 8b. The majority of infections are clustered in the younger age cohort. In Scenario 2, where viral clearance is complete, infections are also mostly concentrated in the younger age group, as shown in Fig. 8c. Compared with these two extremes, our model (p = 0.9) shows that incomplete viral clearance leads to a typical bimodal age distribution, which is more consistent with the observed clinical pattern.

**Fig. 8:**
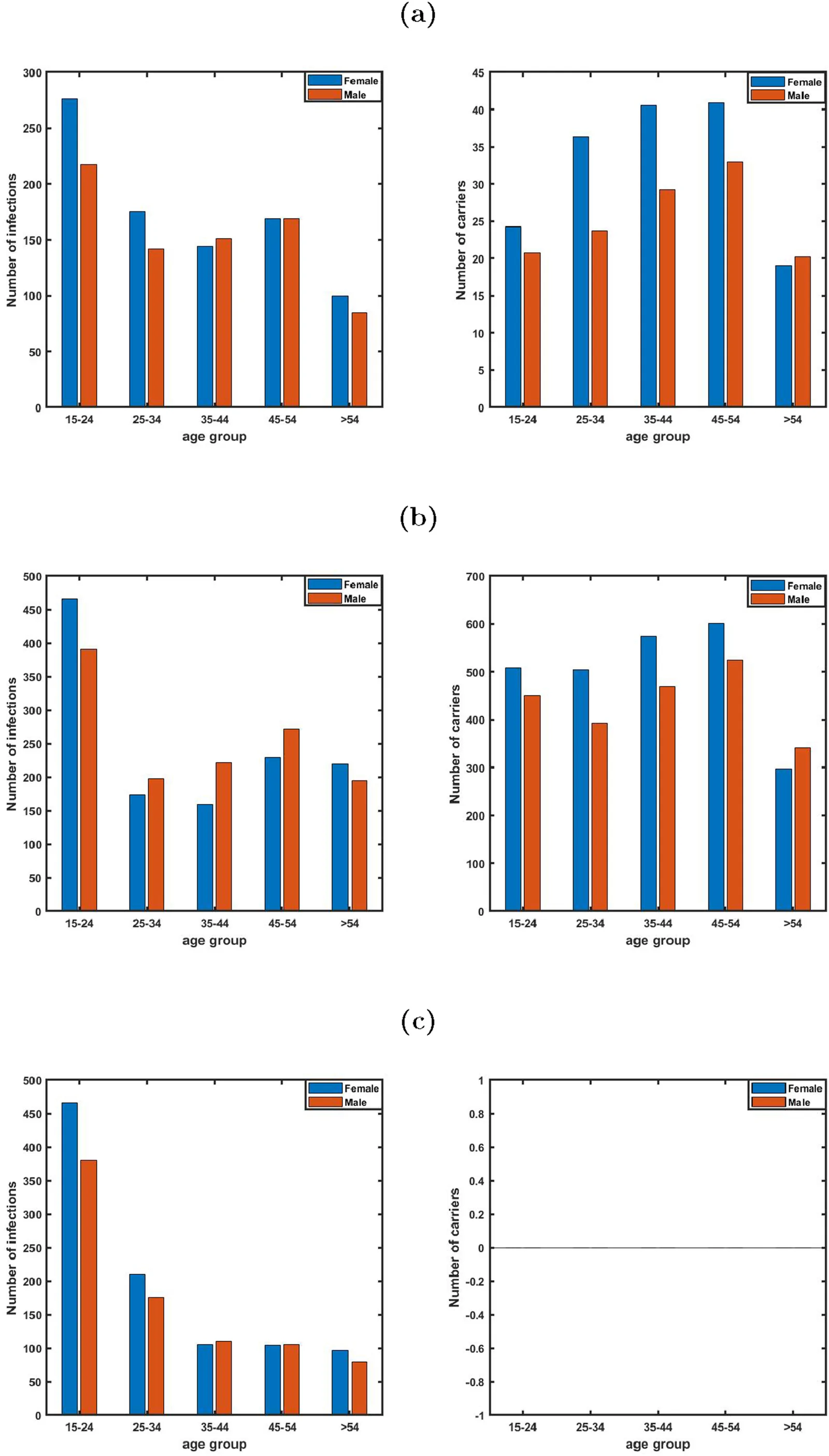
Panels **(a)**-**(c)** show the age-group distributions after 6 years. In each panel, the left subfigure shows the distribution of the number of infections by age group, and the right subfigure shows the distribution of the number of virus carriers by age group. Panel **(a)** corresponds to partial viral clearance, panel **(b)** to no viral clearance, and panel **(c)** to complete viral clearance.

Fig. 9a shows that the effect of the male reactivation rate on $R_{0}$ is slightly stronger than that of the female reactivation rate, and the viral reactivation rate in the younger age group has a greater impact on $R_{0}$, which is consistent with the sensitivity analysis results in Fig. 5 and Table 4. Simultaneously reducing the viral reactivation rates $\alpha_{2}^{f}$, $\alpha_{3}^{f}$, $\alpha_{2}^{m}$, and $\alpha_{3}^{m}$ by the same factor decreases $R_{0}$, but does not reduce it below 1, which is also consistent with Fig. 9b. This indicates that reducing reactivation alone is insufficient for HPV control and that additional intervention measures are needed.

**Fig. 9:**
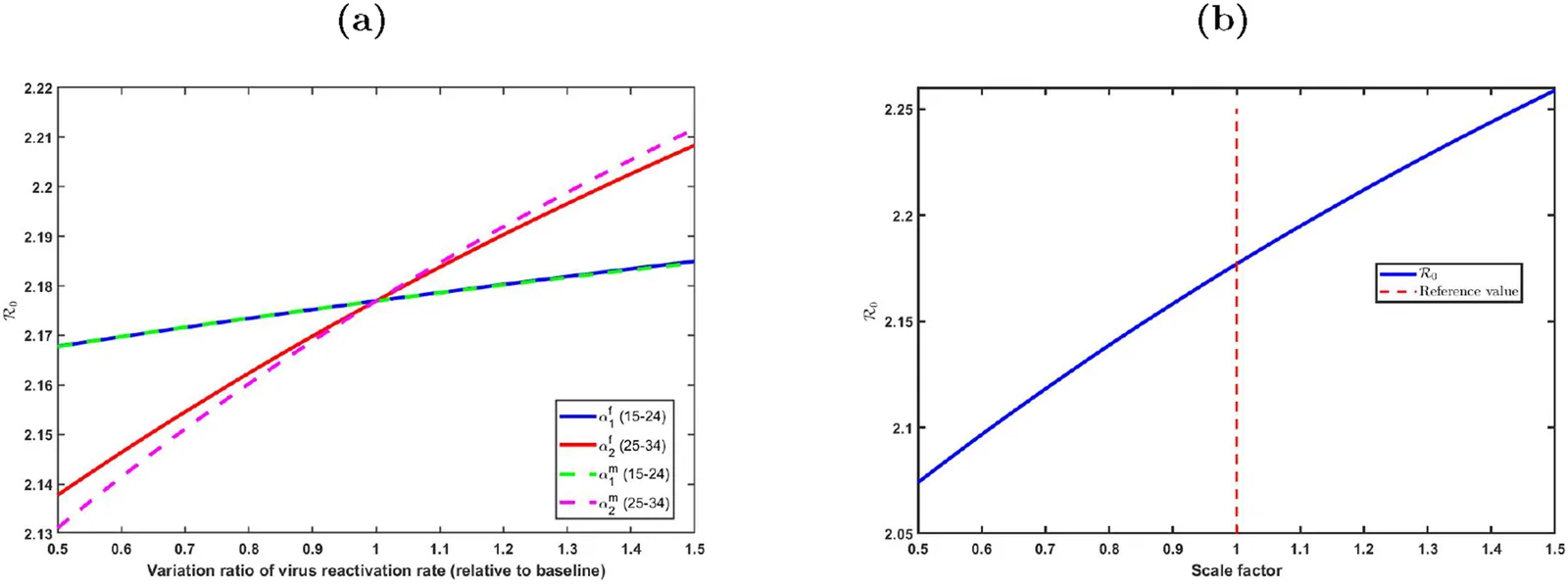
The reference values are $\alpha_{1}^{f}=0.04$, $\alpha_{2}^{f}=0.05$, $\alpha_{1}^{m}=0.06$, and $\alpha_{2}^{m}=0.07$. Panel **(a)** shows how the basic reproduction number varies with these four viral reactivation rates. Panel **(b)** shows how the basic reproduction number varies with the same four viral reactivation rates under a common scaling factor.

Fig. 10a shows that increasing the viral reactivation rates in both sexes leads to a larger $R_{0}$. The similar steepness of the surface in both parameter directions indicates that $R_{0}$ has comparable sensitivity to the reactivation rates of females and males. Similar qualitative patterns are observed for the effects of transmission probabilities and age-specific reactivation rates on $R_{0}$. Notably, the increase in $R_{0}$ shown in Fig. 10c is greater than that in Fig. 10a and b, suggesting that transmission probabilities are more influential determinants of $R_{0}$. Similar conclusions can also be drawn from Fig. 10d. Therefore, interventions that reduce transmission probabilities should be prioritized, while measures targeting viral reactivation should be viewed as important complementary strategies. Overall, Fig. 10 suggests that reducing either transmission rates or reactivation rates can lower $R_{0}$, and that combining these two types of interventions leads to stronger epidemic control.

**Fig. 10:**
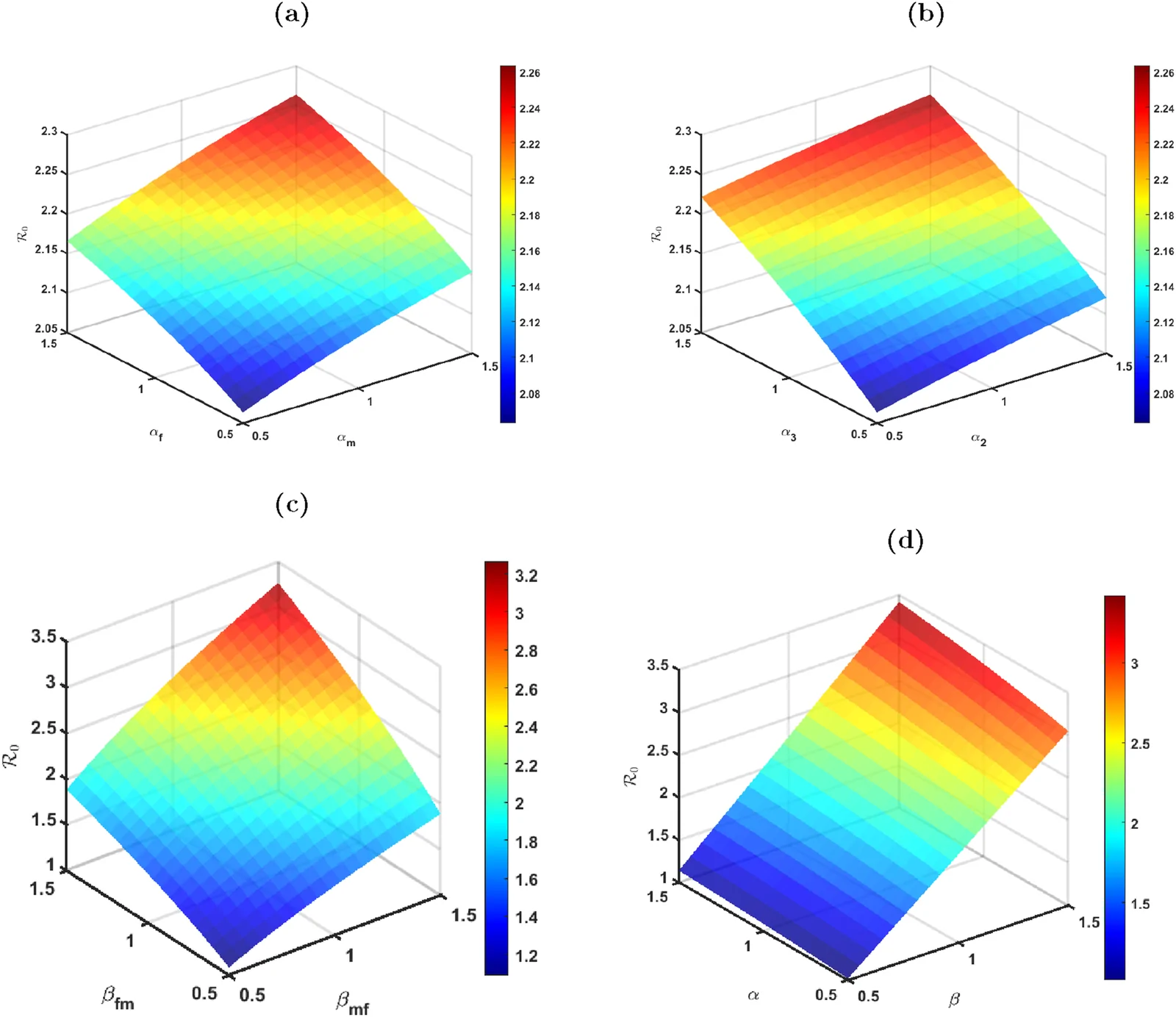
Panel **(a)** shows the impact of sex-specific viral reactivation rates in the 25-44 year age range, namely $\alpha_{f}=\left(\alpha_{2}^{f},\alpha_{3}^{f}\right)$ and $\alpha_{m}=\left(\alpha_{2}^{m},\alpha_{3}^{m}\right)$, on $R_{0}$. Panel **(b)** shows the impact of viral reactivation rates in the first two age groups (25-44 years), namely $\alpha_{2}=\left(\alpha_{2}^{f},\alpha_{2}^{m}\right)$ and $\alpha_{3}=\left(\alpha_{3}^{f},\alpha_{3}^{m}\right)$, on $R_{0}$. Panel **(c)** shows the impact of the transmission probabilities *β* = (*β*_*fm*_, *β*_*mf*_) on $R_{0}$. Panel **(d)** shows the combined impact of sex-specific viral reactivation rates in the first two age groups, *α* = (*α*_*f*_, *α*_*m*_), and transmission probabilities *β* = (*β*_*fm*_, *β*_*mf*_) on $R_{0}$.

The parameter values used in these intervention scenarios were manually selected and adjusted for R<1. Fig. 11 further shows that, under a single intervention, the transmission rate must be reduced by 55% in order to bring $R_{0}$ below 1. In contrast, reducing only the viral reactivation rate or increasing the full recovery rate is insufficient to control HPV transmission. Combined interventions are more effective. For example, HPV prevalence can be controlled by reducing the transmission rate by 53% together with reducing the viral reactivation rate in the young group by 40%, or by reducing the transmission rate by 50% together with reducing the viral reactivation rate by 80%. These results show a clear trade-off: if the viral reactivation rate is reduced only slightly, then the transmission rate must be reduced more substantially; conversely, if the viral reactivation rate is reduced markedly, then a smaller reduction in the transmission rate is sufficient. This finding supports the use of integrated intervention strategies that simultaneously target transmission and reactivation. The studies by Jing et al.(Jing et al. (2024)) and Jia et al.(Jia et al. (2026)) have a similar conclusion: effective epidemic control cannot rely on a single intervention. It requires strategies that match how the disease spreads, such as combining treatment, vaccination, and targeted behavioral changes to reduce disease transmission.

**Fig. 11:**
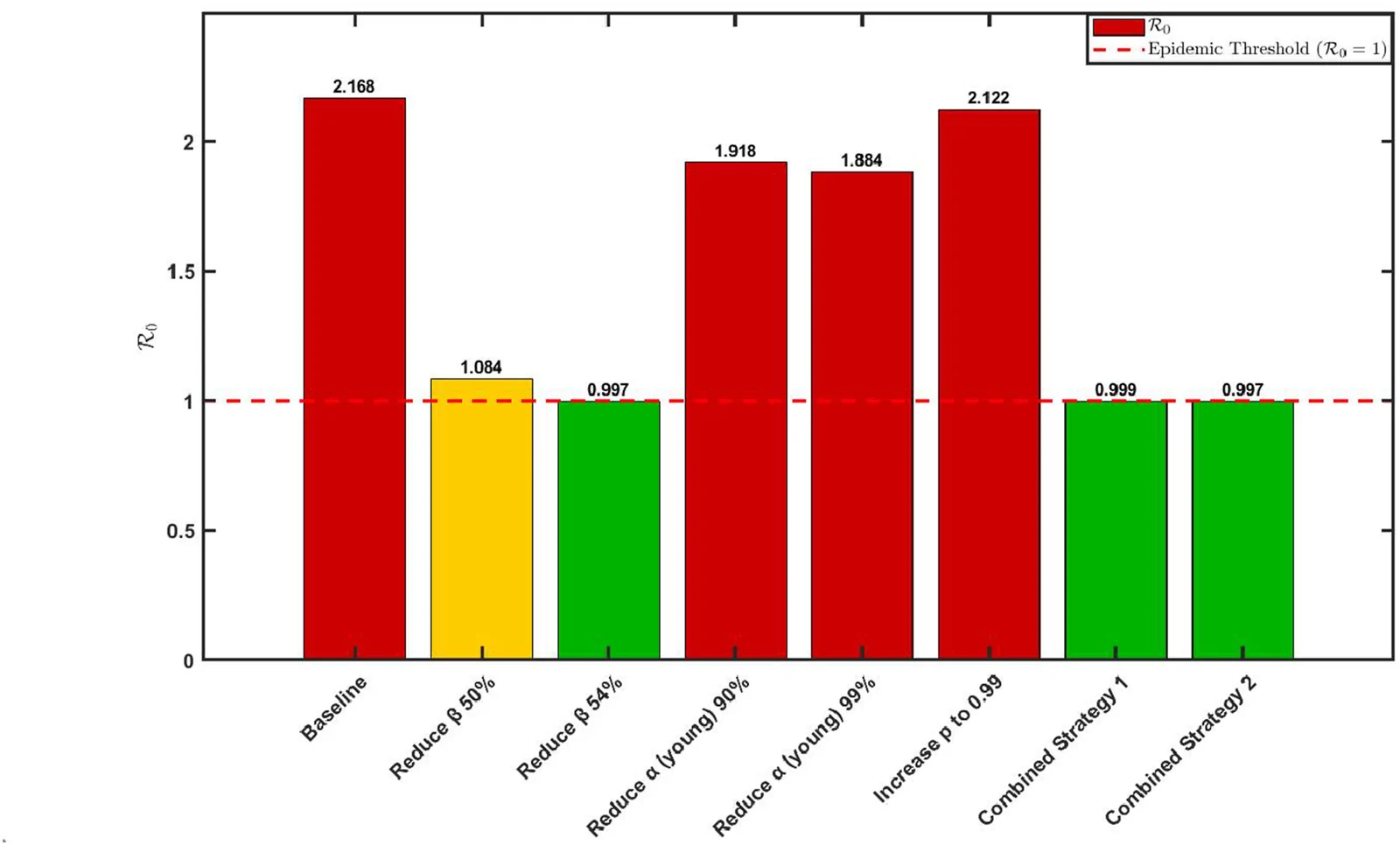
Effect of different intervention strategies on the basic reproduction number $R_{0}$. In Combined Strategy 1, the transmission rate is reduced by 52% and the viral reactivation rate in the young group (25-44 years) is reduced by 37%. In Combined Strategy 2, the transmission rate is reduced by 48% and the viral reactivation rate in the young group is reduced by 92%. The red dashed line represents the threshold $R_{0}=1$.

## 5 Conclusion and discussion

In this paper, we formulated and analyzed a discrete age-structured SICR model with two-sex transmission and viral reactivation to study the transmission dynamics of HPV in a heterosexual population. Unlike many previous HPV models, which assume homogeneous mixing or neglect the role of latent carriage and reactivation (Brouwer et al., 2015; Cody et al., 2021; Gao et al., 2022; Saldana et al., 2020), our framework explicitly incorporates age-specific and sex-specific heterogeneity in viral reactivation. This allows us to investigate how these factors shape both the basic reproduction number $R_{0}$ and the long-term prevalence of infection. Explicit formulas for the reproduction number were derived for the one-age-group and two-age-group cases, together with their biological interpretations. We also established the existence and stability of the disease-free equilibrium. Owing to the complexity of the full model, we proved only the existence of the endemic equilibrium analytically, while its existence and stability were further supported numerically. Overall, the model provides a theoretical framework for understanding the multi-scale transmission dynamics of HPV and a quantitative tool for exploring possible intervention strategies.

One important insight from the model is that latent carriage together with viral reactivation can substantially influence the long-term persistence of HPV. The numerical results show that the model reproduces a bimodal age pattern of infection, with relatively high prevalence in younger (15-24 age group) and older (45-54 age group) age groups. This suggests that HPV burden at older ages may not be explained solely by recent transmission, but may also reflect the contribution of persistent carriage and later viral reactivation. In this sense, the model supports the biological view that HPV persistence is not simply a short-term infection process, but may involve long-term within-host viral maintenance followed by renewed infectious activity. The simulated infection dynamics in both females and males exhibit three stages, namely rapid increase, slower long-term growth, and eventual stabilization, reflecting the combined effects of transmission, immune clearance, aging, and reactivation.

The sensitivity analysis further shows that viral reactivation rates are important drivers of both $R_{0}$ and endemic prevalence. In particular, reactivation rates in younger age groups exert the strongest influence on $R_{0}$, indicating that we should prioritize controlling the viral reactivation rate in the younger age groups. By contrast, reactivation in older age groups appears to have a stronger effect on long-term prevalence, especially among older women. This suggests that the epidemiological roles of reactivation may differ by age: in younger groups, reactivation contributes more strongly to transmission potential, whereas in older groups it contributes more strongly to persistent infection burden. The positive association between reactivation in older women and overall prevalence (Fig. 6b) may also be biologically consistent with reduced immune control at older ages, although this mechanism would require further experimental confirmation.

The model also provides insight into sex-specific roles in HPV transmission. Our simulations suggest that females may contribute more strongly to the long-term latent reservoir of infection (Fig. 8a), while male infection and reactivation in younger age groups may have particularly strong effects on transmission intensity. This does not imply that one sex alone drives the epidemic; rather, it highlights the strongly coupled nature of two-sex transmission. Indeed, the sensitivity analysis shows that reactivation in one sex can influence prevalence in the other, emphasizing that HPV control should be viewed as a coupled population process rather than as two separate epidemics. This is also consistent with the model-based observation that interventions directed at males can produce meaningful indirect benefits for females.

Another biologically relevant finding is the role of carrier infectivity. The comparative simulations with different values of *σ* show that when carriers are more infectious, both the infectious and carrier populations increase across age groups and in both sexes, with particularly noticeable effects in older populations. This indicates that even a partially infectious carrier class may substantially amplify long-term transmission. In other words, carriers may not simply represent a passive epidemiological reservoir, but may actively contribute to maintaining HPV circulation, especially when viral clearance is incomplete. The model therefore suggests that the infectivity of latent or carrier states is an important quantity in determining the long-term epidemic burden.

From a public health perspective, the model suggests that effective HPV control should not focus solely on reducing immediate transmission, but should also account for biological mechanisms that sustain infection over long time scales. In particular, the strong influence of reactivation in younger age groups on $R_{0}$ suggests that early intervention, including vaccination and prevention efforts targeting adolescents and young adults, may be especially effective in reducing transmission. The model therefore supports the idea that HPV prevention should be age-specific as well as sex-inclusive. It also suggests that combined strategies targeting both transmission and reactivation may be more effective than strategies focusing on only one mechanism.

The intervention analysis reinforces this conclusion. In the model, reducing the transmission rate alone requires a substantial reduction before $R_{0}$ can be brought below 1, whereas reducing reactivation alone is insufficient for elimination. By contrast, combined reductions in transmission and reactivation achieve stronger epidemic control. This suggests that integrated strategies, such as vaccination, safer-sex education, screening, and measures that reduce persistent infection or reactivation, may be more effective than any single intervention in isolation. Although the specific numerical targets obtained here depend on model assumptions and should not be interpreted as direct policy prescriptions, they provide useful comparative evidence on how different mechanisms contribute to epidemic control.

There are several limitations of this study. First, the model assumes homogeneous mixing within and between age groups and therefore does not capture more complex sexual network structures such as partnership duration, concurrency, or contact heterogeneity. Second, although some parameters were obtained from published studies and others were fitted to available data, several remain difficult to estimate accurately. Third, the present model focuses on HPV transmission prior to vaccination and does not explicitly incorporate vaccine-induced protection. In addition, the model does not distinguish among different HPV types or account for behavioral changes over time. Including type-specific transmission, vaccination, and time-dependent behavior would improve the biological realism of the framework. Finall, the model fitting was based on only five time-specific observations per age group, which may limit the precision and robustness of the estimated parameters. We will use more comprehensive age-specific longitudinal data in future studies, when available, to refine parameter estimation and further validate the model.

The novelty of this study lies in the development of an HPV transmission-dynamic model that incorporates heterogeneity by both gender and age. This is the first modelling framework to systematically quantify the contribution of viral reactivation to the overall HPV infection burden. Unlike many existing models that treat HPV infection as a one-time event or just focus on infections, our model introduces an explicit reactivation compartment. This extension addresses a key limitation of conventional SICR structures, which do not distinguish endogenous viral reactivation from exogenous reinfection. The proposed framework therefore provides a useful mathematical approach for investigating the potential mechanisms underlying the second peak in HPV incidence observed later in adulthood.

In summary, this study develops an SICR framework that integrates age structure, two-sex transmission, and viral reactivation to investigate the long-term dynamics of HPV. The results suggest that viral reactivation is an important mechanism sustaining HPV endemicity, that the interaction between age structure and reactivation may help explain bimodal infection patterns, and that sex-specific and age-specific heterogeneity should be taken into account when designing control strategies. By combining mathematical analysis with numerical investigation, this work provides a useful framework for understanding persistent HPV transmission and for evaluating intervention strategies aimed at long-term control.

## CRediT authorship contribution statement

**Siqi Shao:** Writing – original draft, Software, Methodology, Formal analysis, Data curation, Conceptualization. **Ting Guo:** Writing – review & editing, Methodology. **Shasha Gao:** Writing – review & editing, Supervision, Methodology, Conceptualization.

## Data availability

All relevant data used in this study are referenced in the article, and the fitting data are included in the additional file.

## Declaration of competing interests

The authors declare that they have no known competing financial interests or personal relationships that could have appeared to influence the work reported in this paper.

## Appendix A Proof of the well-posedness of system (1)

For convenience, we first recall a theorem from Thieme (2018). Let $R_{+}^{n}={\left(\left[0,+\infty \right)\right)}^{n}$ denote the cone of nonnegative vectors in $R^{n}$. Let $F:R_{+}^{n+1}\to R^{n}$,

$$F\left(t,x\right)=\left(F_{1}\left(t,x\right),\ldots ,F_{n}\left(t,x\right)\right),\;x=\left(x_{1},\ldots ,x_{n}\right),$$

be locally Lipschitz, and suppose that *F*_*j*_(*t*, *x*) ≥ 0 whenever *t* ≥ 0, $x\in R_{+}^{n}$, and *x*_*j*_ = 0. Then, for every $x^{0}\in R_{+}^{n}$, there exists a unique solution of *x*′ = *F*(*t*, *x*) with *x*(0) = *x*^0^, defined on some interval $\left(\left[0,b\right)\right)$, where *b* > 0. If *b* < *∞*, then

$$\lim_{t\to b}\sup\;\overset{n}{\underset{j=1}{\sum }}x_{j}\left(t\right)=\infty .$$

We now use this theorem to prove that system (1) is well posed. First, we show that the region

$$\begin{array}{ll} D= & \left\{\left(S_{1}^{f},\ldots ,S_{n}^{f},I_{1}^{f},\ldots ,I_{n}^{f},C_{1}^{f},\ldots ,C_{n}^{f},R_{1}^{f},\ldots ,R_{n}^{f},S_{1}^{m},\ldots ,S_{n}^{m},I_{1}^{m},\ldots ,I_{n}^{m},C_{1}^{m},\ldots ,C_{n}^{m},\right)\right)\\ & \left(\left(R_{1}^{m},\ldots ,R_{n}^{m}\right)\in R_{+}^{8n}:\overset{n}{\underset{j=1}{\sum }}S_{j}^{g}+I_{j}^{g}+C_{j}^{g}+R_{j}^{g}\leq \frac{\Lambda^{g}}{\mu^{g}},\;g\in \left\{f,m\right\}\right\} \end{array}$$

is positively invariant and attracts all solutions of system (1). From

$$\frac{dN^{g}}{dt}=\Lambda^{g}-\mu^{g}N^{g},\;g\in \left\{f,m\right\},$$

we have

$$N^{g}\left(t\right)=\frac{\Lambda^{g}}{\mu^{g}}+\left[N^{g}\left(0\right)-\frac{\Lambda^{g}}{\mu^{g}}\right]e^{-\mu^{g}t},\;g\in \left\{f,m\right\}.$$

Therefore, if $N^{g}\left(0\right)\leq \frac{\Lambda^{g}}{\mu^{g}}$, then $N^{g}\left(t\right)\leq \frac{\Lambda^{g}}{\mu^{g}}$ for all *t* ≥ 0. This shows that *D* is positively invariant.

If $N^{g}\left(0\right)>\frac{\Lambda^{g}}{\mu^{g}}$, then

$$N^{g}\left(t\right)=\frac{\Lambda^{g}}{\mu^{g}}+\left[N^{g}\left(0\right)-\frac{\Lambda^{g}}{\mu^{g}}\right]e^{-\mu^{g}t}>\frac{\Lambda^{g}}{\mu^{g}},$$

and therefore

$$\frac{dN^{g}}{dt}=\Lambda^{g}-\mu^{g}N^{g}<0.$$

Hence, *N*^*g*^(*t*) is strictly decreasing. Since it is bounded below by $\frac{\Lambda^{g}}{\mu^{g}}$, it follows that

$$N^{g}\left(t\right)\to \frac{\Lambda^{g}}{\mu^{g}}\;\text{as}t\to \infty .$$

Thus, if $N^{g}\left(0\right)>\frac{\Lambda^{g}}{\mu^{g}}$, then *N*^*g*^(*t*) approaches $\frac{\Lambda^{g}}{\mu^{g}}$ asymptotically. This shows that the region *D* attracts all solutions in $R_{+}^{8n}$. Therefore, *D* is epidemiologically relevant and positively invariant.

Next, we use Thieme's theorem to prove that for any initial value in *D*, there exists a unique solution of system (1) with values in *D* for all $t\in \left(\left[0,+\infty \right)\right)$. Let

$$x=\left(S_{1}^{f},\ldots ,S_{n}^{f},I_{1}^{f},\ldots ,I_{n}^{f},C_{1}^{f},\ldots ,C_{n}^{f},R_{1}^{f},\ldots ,R_{n}^{f},S_{1}^{m},\ldots ,S_{n}^{m},I_{1}^{m},\ldots ,I_{n}^{m},C_{1}^{m},\ldots ,C_{n}^{m},R_{1}^{m},\ldots ,R_{n}^{m}\right),$$

and define

$$\begin{array}{ll} & F_{1}\left(x\right)=\Lambda^{f}+\omega_{1}^{f}R_{1}^{f}-\lambda_{1}^{f}\left(t\right)S_{1}^{f}-\left(a_{1}^{f}+\mu_{1}^{f}\right)S_{1}^{f},\\ & \vdots \\ & F_{n}\left(x\right)=\omega_{n}^{f}R_{n}^{f}-\lambda_{n}^{f}\left(t\right)S_{n}^{f}-\mu_{n}^{f}S_{n}^{f}+a_{n-1}^{f}S_{n-1}^{f},\\ & F_{n+1}\left(x\right)=\lambda_{1}^{f}\left(t\right)S_{1}^{f}+\alpha_{1}^{f}C_{1}^{f}-\left(\gamma_{1}^{f}+a_{1}^{f}+\mu_{1}^{f}\right)I_{1}^{f},\\ & \vdots \\ & F_{2n}\left(x\right)=\lambda_{n}^{f}\left(t\right)S_{n}^{f}+\alpha_{n}^{f}C_{n}^{f}-\left(\gamma_{n}^{f}+\mu_{n}^{f}\right)I_{n}^{f}+a_{n-1}^{f}I_{n-1}^{f},\\ & F_{2n+1}\left(x\right)=\left(1-p\right)\gamma_{1}^{f}I_{1}^{f}-\left(\alpha_{1}^{f}+a_{1}^{f}+\mu_{1}^{f}\right)C_{1}^{f},\\ & \vdots \\ & F_{3n}\left(x\right)=\left(1-p\right)\gamma_{n}^{f}I_{n}^{f}-\left(\alpha_{n}^{f}+\mu_{n}^{f}\right)C_{n}^{f}+a_{n-1}^{f}C_{n-1}^{f},\\ & F_{3n+1}\left(x\right)=p\gamma_{1}^{f}I_{1}^{f}-\left(\omega_{1}^{f}+a_{1}^{f}+\mu_{1}^{f}\right)R_{1}^{f},\\ & \vdots \\ & F_{4n}\left(x\right)=p\gamma_{n}^{f}I_{n}^{f}-\left(\omega_{n}^{f}+\mu_{n}^{f}\right)R_{n}^{f}+a_{n-1}^{f}R_{n-1}^{f},\\ & \vdots \end{array}$$

For any $x,\bar{x}$, we assume that $S_{1}^{f}<N^{m}$ always holds. Then

$$\begin{array}{ll} \left|F_{1}\left(x\right)-F_{1}\left(\bar{x}\right)\right| & =\left|\lambda_{1}^{f}\left({\bar{S}}_{1}^{f}-S_{1}^{f}\right)+\left({\bar{\lambda }}_{1}^{f}-\lambda_{1}^{f}\right){\bar{S}}_{1}^{f}+\omega_{1}^{f}\left(R_{1}^{f}-{\bar{R}}_{1}^{f}\right)-\left(a_{1}^{f}+\mu_{1}^{f}\right)\left(S_{1}^{f}-{\bar{S}}_{1}^{f}\right)\right|\\ & \leq \left|\lambda_{1}^{f}\left({\bar{S}}_{1}^{f}-S_{1}^{f}\right)\right|+\left|\left({\bar{\lambda }}_{1}^{f}-\lambda_{1}^{f}\right){\bar{S}}_{1}^{f}\right|+\left|\omega_{1}^{f}\left(R_{1}^{f}-{\bar{R}}_{1}^{f}\right)\right|+\left|\left(a_{1}^{f}+\mu_{1}^{f}\right)\left(S_{1}^{f}-{\bar{S}}_{1}^{f}\right)\right|\\ & \leq M_{1}\left|{\bar{S}}_{1}^{f}-S_{1}^{f}\right|+{\bar{S}}_{1}^{f}\left|{\bar{\lambda }}_{1}^{f}-\lambda_{1}^{f}\right|+\omega_{1}^{f}\left|R_{1}^{f}-{\bar{R}}_{1}^{f}\right|+\left(a_{1}^{f}+\mu_{1}^{f}\right)\left|S_{1}^{f}-{\bar{S}}_{1}^{f}\right|\\ & \leq M_{1}\left|{\bar{S}}_{1}^{f}-S_{1}^{f}\right|+\overset{n}{\underset{j=1}{\sum }}\frac{c_{1j}^{fm}\beta_{mf}{\bar{S}}_{1}^{f}}{N_{j}^{m}{\bar{N}}_{j}^{m}}\left|\left({\bar{I}}_{j}^{m}+\sigma {\bar{C}}_{j}^{m}\right)N_{j}^{m}-\left(I_{j}^{m}+\sigma C_{j}^{m}\right){\bar{N}}_{j}^{m}\right|+\omega_{1}^{f}\left|R_{1}^{f}-{\bar{R}}_{1}^{f}\right|\\ & \;+\left(a_{1}^{f}+\mu_{1}^{f}\right)\left|S_{1}^{f}-{\bar{S}}_{1}^{f}\right|\\ & =M_{1}\left|{\bar{S}}_{1}^{f}-S_{1}^{f}\right|+\omega_{1}^{f}\left|R_{1}^{f}-{\bar{R}}_{1}^{f}\right|+\left(a_{1}^{f}+\mu_{1}^{f}\right)\left|S_{1}^{f}-{\bar{S}}_{1}^{f}\right|\\ & \;+\overset{n}{\underset{j=1}{\sum }}\frac{c_{1j}^{fm}\beta_{mf}{\bar{S}}_{1}^{f}}{N_{j}^{m}{\bar{N}}_{j}^{m}}\left|\left(I_{j}^{m}+\sigma C_{j}^{m}\right)\left({\bar{N}}_{j}^{m}-N_{j}^{m}\right)+\left(I_{j}^{m}+\sigma C_{j}^{m}-{\bar{I}}_{j}^{m}-\sigma {\bar{C}}_{j}^{m}\right)N_{j}^{m}\right|\\ & \leq M_{1}\left|{\bar{S}}_{1}^{f}-S_{1}^{f}\right|+\omega_{1}^{f}\left|R_{1}^{f}-{\bar{R}}_{1}^{f}\right|+\left(a_{1}^{f}+\mu_{1}^{f}\right)\left|S_{1}^{f}-{\bar{S}}_{1}^{f}\right|+\\ & \;\overset{n}{\underset{j=1}{\sum }}\frac{c_{1j}^{fm}\beta_{mf}{\bar{S}}_{1}^{f}\left(I_{j}^{m}+\sigma C_{j}^{m}\right)}{N_{j}^{m}{\bar{N}}_{j}^{m}}\left|{\bar{N}}_{j}^{m}-N_{j}^{m}\right|+\overset{n}{\underset{j=1}{\sum }}\frac{c_{1j}^{fm}\beta_{mf}{\bar{S}}_{1}^{f}}{{\bar{N}}_{j}^{m}}\left|I_{j}^{m}+\sigma C_{j}^{m}-{\bar{I}}_{j}^{m}-\sigma {\bar{C}}_{j}^{m}\right|\\ & \leq M_{1}\left|{\bar{S}}_{1}^{f}-S_{1}^{f}\right|+\omega_{1}^{f}\left|R_{1}^{f}-{\bar{R}}_{1}^{f}\right|+\left(a_{1}^{f}+\mu_{1}^{f}\right)\left|S_{1}^{f}-{\bar{S}}_{1}^{f}\right|+M_{1}\left|N_{j}^{m}-{\bar{N}}_{j}^{m}\right|\\ & \;+\overset{n}{\underset{j=1}{\sum }}c_{1j}^{fm}\beta_{mf}\left|I_{j}^{m}+\sigma C_{j}^{m}-{\bar{I}}_{j}^{m}-\sigma {\bar{C}}_{j}^{m}\right|\\ & \leq \left(M_{1}+\omega_{1}^{f}+a_{1}^{f}+\mu_{1}^{f}\right)\left(\left|S_{1}^{f}-{\bar{S}}_{1}^{f}\right|+\left|R_{1}^{f}-{\bar{R}}_{1}^{f}\right|\right)+M_{1}\left|N_{j}^{m}-{\bar{N}}_{j}^{m}\right|+\\ & \;\overset{n}{\underset{j=1}{\sum }}c_{1j}^{fm}\beta_{mf}\left|I_{j}^{m}-{\bar{I}}_{j}^{m}\right|+\overset{n}{\underset{j=1}{\sum }}c_{1j}^{fm}\beta_{mf}\sigma \left|C_{j}^{m}-{\bar{C}}_{j}^{m}\right|\\ & \leq M_{2}\|x-\bar{x}\|, \end{array}$$

where

$$M_{1}=\overset{n}{\underset{j=1}{\sum }}c_{1j}^{fm}\beta_{mf},\;M_{2}=\max\left\{M_{1}+\omega_{1}^{f}+a_{1}^{f}+\mu_{1}^{f},c_{1j}^{fm}\beta_{mf}\right\},\;j=1,2,\ldots ,n,$$

and

$$\begin{array}{ll} \left\|x-\bar{x}\right\| & =\left|S_{1}^{f}-{\bar{S}}_{1}^{f}\right|+\cdots +\left|S_{n}^{f}-{\bar{S}}_{n}^{f}\right|+\left|I_{1}^{f}-{\bar{I}}_{1}^{f}\right|+\cdots +\left|I_{n}^{f}-{\bar{I}}_{n}^{f}\right|+\left|C_{1}^{f}-{\bar{C}}_{1}^{f}\right|+\cdots +\left|C_{n}^{f}-{\bar{C}}_{n}^{f}\right|\\ & \;+\left|R_{1}^{f}-{\bar{R}}_{1}^{f}\right|+\cdots +\left|R_{n}^{f}-{\bar{R}}_{n}^{f}\right|+\left|S_{1}^{m}-{\bar{S}}_{1}^{m}\right|+\cdots +\left|S_{n}^{m}-{\bar{S}}_{n}^{m}\right|+\left|I_{1}^{m}-{\bar{I}}_{1}^{m}\right|+\cdots +\left|I_{n}^{m}-{\bar{I}}_{n}^{m}\right|\\ & \;+\left|C_{1}^{m}-{\bar{C}}_{1}^{m}\right|+\cdots +\left|C_{n}^{m}-{\bar{C}}_{n}^{m}\right|+\left|R_{1}^{m}-{\bar{R}}_{1}^{m}\right|+\cdots +\left|R_{n}^{m}-{\bar{R}}_{n}^{m}\right|. \end{array}$$

By similar arguments, we can show that the other components of *F*(*x*) are also locally Lipschitz in *x*. Hence, there exists some *M* > 0 such that $\left\|F\left(x\right)-F\left(\bar{x}\right)\|\leq M\|x-\bar{x}\right\|$. Therefore, *F*(*x*) is locally Lipschitz.

If $S_{1}^{f}=0$, then $F_{1}\left(x\right)=\Lambda^{f}+\omega_{1}^{f}R_{1}^{f}\geq 0$ whenever *t* ≥ 0 and $x\in R_{+}^{8n}$. Similarly, we have *F*_*k*_(*x*) ≥ 0 whenever *t* ≥ 0, $x\in R_{+}^{8n}$, and *k* = 2, …, 8*n*. Therefore, by Thieme's theorem, for every *x*^0^ ∈ *D*, there exists a unique solution of *x*′ = *F*(*x*) on some interval $\left(\left[0,b\right)\right)$, with *b* > 0. Moreover, since

$$\overset{8n}{\underset{j=1}{\sum }}x_{j}\left(t\right)=N\left(t\right)\leq \max\left\{N\left(0\right),\frac{\Lambda^{f}}{\mu^{f}}+\frac{\Lambda^{m}}{\mu^{m}}\right\}<\infty ,$$

we have

$$\lim_{t\to b}\sup\;\|x\left(t\right)\|<\infty .$$

By Thieme's theorem again, it follows that *b* = *∞*. Thus, the model is both epidemiologically and mathematically well posed.

## Appendix B The basic reproduction numbers for two special cases

Case 1 Basic reproduction number within one age group

In this case, system (1) reduces to

(5) $$\left\{\begin{array}{l} \frac{dS_{1}^{g}}{dt}=\Lambda^{g}+\omega_{1}^{g}R_{1}^{g}-\lambda_{1}^{g}\left(t\right)S_{1}^{g}-\mu_{1}^{g}S_{1}^{g},\\ \frac{dI_{1}^{g}}{dt}=\lambda_{1}^{g}\left(t\right)S_{1}^{g}+\alpha_{1}^{g}C_{1}^{g}-\left(\gamma_{1}^{g}+\mu_{1}^{g}\right)I_{1}^{g},\\ \frac{dC_{1}^{g}}{dt}=\left(1-p\right)\gamma_{1}^{g}I_{1}^{g}-\left(\alpha_{1}^{g}+\mu_{1}^{g}\right)C_{1}^{g},\\ \frac{dR_{1}^{g}}{dt}=p\gamma_{1}^{g}I_{1}^{g}-\left(\omega_{1}^{g}+\mu_{1}^{g}\right)R_{1}^{g}. \end{array}\right)\;g\in \left\{f,m\right\}$$

The forces of infection are given by

$$\lambda_{1}^{f}\left(t\right)=c_{11}^{fm}\frac{\beta_{mf}\left(I_{1}^{m}\left(t\right)+\sigma \cdot C_{1}^{m}\left(t\right)\right)}{N_{1}^{m}\left(t\right)},\;\lambda_{1}^{m}\left(t\right)=c_{11}^{mf}\frac{\beta_{fm}\left(I_{1}^{f}\left(t\right)+\sigma \cdot C_{1}^{f}\left(t\right)\right)}{N_{1}^{f}\left(t\right)}.$$

We consider the infected compartments

$$x=\left(I_{1}^{f},C_{1}^{f},I_{1}^{m},C_{1}^{m}\right),$$

and again apply the next-generation matrix method to determine the basic reproduction number for this one-age-group system. Note that the DFE for the one-age-group case is

$$E_{0}=\left(S_{1}^{f0},S_{1}^{m0},0,0,0,0,0,0\right)$$

with $S_{1}^{f0}=\frac{\Lambda^{f}}{a_{1}^{f}+\mu_{1}^{f}}$ and $S_{1}^{m0}=\frac{\Lambda^{m}}{a_{1}^{m}+\mu_{1}^{m}}$. Writing the system in terms of the matrix of new infections *F* and the matrix of transitions *V*, we obtain

$$F\overset{△}{=}\left(\begin{array}{ll} 0 & F_{fm}\\ F_{mf} & 0 \end{array}\right)={\left(\begin{array}{llll} 0 & 0 & F_{13} & F_{14}\\ 0 & 0 & 0 & 0\\ F_{31} & F_{32} & 0 & 0\\ 0 & 0 & 0 & 0 \end{array}\right)}_{4\times 4},\;V={\left(\begin{array}{ll} V^{f} & 0\\ 0 & V^{m} \end{array}\right)}_{4\times 4},$$

where

$$\begin{array}{l} F_{13}=c_{11}^{fm}\frac{\beta_{mf}}{N_{1}^{m0}}S_{1}^{f0},\;F_{31}=c_{11}^{mf}\frac{\beta_{fm}}{N_{1}^{f0}}S_{1}^{m0},\\ V^{g}={\left(\begin{array}{ll} V_{II}^{g} & V_{IC}^{g}\\ V_{CI}^{g} & V_{CC}^{g} \end{array}\right)}_{2\times 2}={\left(\begin{array}{ll} \gamma_{1}^{g}+\mu_{1}^{g} & -\alpha_{1}^{g}\\ -\left(1-p\right)\gamma_{1}^{g} & \alpha_{1}^{g}+\mu_{1}^{g} \end{array}\right)}_{2\times 2}, \end{array}$$

and

$$V^{-1}={\left(\begin{array}{llll} \frac{\alpha_{1}^{f}+\mu_{1}^{f}}{D^{f}} & \frac{\alpha_{1}^{f}}{D^{f}} & 0 & 0\\ \frac{\left(1-p\right)\gamma_{1}^{f}}{D^{f}} & \frac{\gamma_{1}^{f}+\mu_{1}^{f}}{D^{f}} & 0 & 0\\ 0 & 0 & \frac{\alpha_{1}^{m}+\mu_{1}^{m}}{D^{m}} & \frac{\alpha_{1}^{m}}{D^{m}}\\ 0 & 0 & \frac{\left(1-p\right)\gamma_{1}^{m}}{D^{m}} & \frac{\gamma_{1}^{m}+\mu_{1}^{m}}{D^{m}} \end{array}\right)}_{4\times 4},$$

where

$$D^{g}=\frac{1}{\left(\gamma_{1}^{g}+\mu_{1}^{g}\right)\left(\alpha_{1}^{g}+\mu_{1}^{g}\right)-\left(1-p\right)\gamma_{1}^{g}\alpha_{1}^{g}},g\in \left\{f,m\right\}.$$

Then the next-generation matrix is

$$FV^{-1}=\left(\begin{array}{llll} 0 & 0 & X_{3} & X_{4}\\ 0 & 0 & 0 & 0\\ X_{1} & X_{2} & 0 & 0\\ 0 & 0 & 0 & 0 \end{array}\right),$$

where

$$\begin{array}{ll} & X_{1}=c_{11}^{mf}\frac{\beta_{fm}}{N_{1}^{f0}}S_{1}^{m0}\frac{\alpha_{1}^{f}+\mu_{1}^{f}}{D^{f}}+\sigma c_{11}^{mf}\frac{\beta_{fm}}{N_{1}^{f0}}S_{1}^{m0}\frac{\left(1-p\right)\gamma_{1}^{f}}{D^{f}},\\ & X_{2}=c_{11}^{mf}\frac{\beta_{fm}}{N_{1}^{f0}}S_{1}^{m0}\frac{\alpha_{1}^{f}}{D^{f}}+\sigma c_{11}^{mf}\frac{\beta_{fm}}{N_{1}^{f0}}S_{1}^{m0}\frac{\gamma_{1}^{f}+\mu_{1}^{f}}{D^{f}},\\ & X_{3}=c_{11}^{fm}\frac{\beta_{mf}}{N_{m}^{f0}}S_{1}^{f0}\frac{\alpha_{1}^{m}+\mu_{1}^{m}}{D^{m}}+\sigma c_{11}^{fm}\frac{\beta_{mf}}{N_{1}^{m0}}S_{1}^{f0}\frac{\left(1-p\right)\gamma_{1}^{m}}{D^{m}},\\ & X_{4}=c_{11}^{fm}\frac{\beta_{mf}}{N_{1}^{m0}}S_{1}^{f0}\frac{\alpha_{1}^{m}}{D^{m}}+\sigma c_{11}^{fm}\frac{\beta_{mf}}{N_{1}^{m0}}S_{1}^{f0}\frac{\gamma_{1}^{m}+\mu_{1}^{m}}{D^{m}}. \end{array}$$

Therefore, the basic reproduction number for one age group is

$$R_{0}^{11}\equiv \sqrt{R_{0}^{f1}R_{0}^{m1}},$$

with

$$\begin{array}{ll} & R_{0}^{m1}=c_{11}^{fm}\frac{\beta_{mf}}{N_{1}^{m0}}S_{1}^{f0}\frac{\alpha_{1}^{m}+\mu_{1}^{m}}{D^{m}}+\sigma c_{11}^{fm}\frac{\beta_{mf}}{N_{1}^{m0}}S_{1}^{f0}\frac{\left(1-p\right)\gamma_{1}^{m}}{D^{m}},\\ & R_{0}^{f1}=c_{11}^{mf}\frac{\beta_{fm}}{N_{1}^{\mathrm{mf0}}}S_{1}^{m0}\frac{\alpha_{1}^{f}+\mu_{1}^{f}}{D^{f}}+\sigma c_{11}^{mf}\frac{\beta_{fm}}{N_{1}^{f0}}S_{1}^{m0}\frac{\left(1-p\right)\gamma_{1}^{f}}{D^{f}}. \end{array}$$

Case 2 Basic reproduction number between two age groups

In this case, system (1) reduces to

$$\;\left\{\begin{array}{l} \frac{dS_{1}^{g}}{dt}=\Lambda^{g}+\omega_{1}^{g}R_{1}^{g}-\lambda_{1}^{g}\left(t\right)S_{1}^{g}-\left(a_{1}^{g}+\mu_{1}^{g}\right)S_{1}^{g},\\ \frac{dI_{1}^{g}}{dt}=\lambda_{1}^{g}\left(t\right)S_{1}^{g}+\alpha_{1}^{g}C_{1}^{g}-\left(\gamma_{1}^{g}+a_{1}^{g}+\mu_{1}^{g}\right)I_{1}^{g},\\ \frac{dC_{1}^{g}}{dt}=\left(1-p\right)\gamma_{1}^{g}I_{1}^{g}-\left(\alpha_{1}^{g}+a_{1}^{g}+\mu_{1}^{g}\right)C_{1}^{g},\\ \frac{dR_{1}^{g}}{dt}=p\gamma_{1}^{g}I_{1}^{g}-\left(\omega_{1}^{g}+a_{1}^{g}+\mu_{1}^{g}\right)R_{1}^{g},\\ \frac{dS_{2}^{g}}{dt}=\omega_{2}^{g}R_{2}^{g}-\lambda_{2}^{g}\left(t\right)S_{2}^{g}-\mu_{2}^{g}S_{2}^{g}+a_{1}^{g}S_{1}^{g},\\ \frac{dI_{2}^{g}}{dt}=\lambda_{2}^{g}\left(t\right)S_{2}^{g}+\alpha_{2}^{g}C_{2}^{g}-\left(\gamma_{2}^{g}+\mu_{2}^{g}\right)I_{2}^{g}+a_{1}^{g}I_{1}^{g},\\ \frac{dC_{2}^{g}}{dt}=\left(1-p\right)\gamma_{2}^{g}I_{2}^{g}-\left(\alpha_{2}^{g}+\mu_{2}^{g}\right)C_{2}^{g}+a_{1}^{g}C_{1}^{g},\\ \frac{dR_{2}^{g}}{dt}=p\gamma_{2}^{g}I_{2}^{g}-\left(\omega_{2}^{g}+\mu_{2}^{g}\right)R_{2}^{g}+a_{1}^{g}R_{1}^{g} \end{array}\right)$$

where the female forces of infection are

$$\lambda_{1}^{f}\left(t\right)=\overset{2}{\underset{j=1}{\sum }}c_{1j}^{fm}\frac{\beta_{mf}\left(I_{j}^{m}\left(t\right)+\sigma \cdot C_{j}^{m}\left(t\right)\right)}{N_{j}^{m}\left(t\right)},\;\lambda_{2}^{f}\left(t\right)=\overset{2}{\underset{j=1}{\sum }}c_{2j}^{fm}\frac{\beta_{mf}\left(I_{j}^{m}\left(t\right)+\sigma \cdot C_{j}^{m}\left(t\right)\right)}{N_{j}^{m}\left(t\right)},$$

and the male forces of infection are

$$\lambda_{1}^{m}\left(t\right)=\overset{2}{\underset{j=1}{\sum }}c_{1j}^{mf}\frac{\beta_{fm}\left(I_{j}^{f}\left(t\right)+\sigma \cdot C_{j}^{f}\left(t\right)\right)}{N_{j}^{f}\left(t\right)},\;\lambda_{2}^{m}\left(t\right)=\overset{2}{\underset{j=1}{\sum }}c_{2j}^{mf}\frac{\beta_{fm}\left(I_{j}^{f}\left(t\right)+\sigma \cdot C_{j}^{f}\left(t\right)\right)}{N_{j}^{f}\left(t\right)}.$$

We consider the infected compartments

$$\left(I_{1}^{f},I_{2}^{f},C_{1}^{f},C_{2}^{f},I_{1}^{m},I_{2}^{m},C_{1}^{m},C_{2}^{m}\right),$$

and write the system in terms of the matrix of new infections *F* and the matrix of transitions *V*:

$$F\overset{△}{=}\left(\begin{array}{ll} 0 & F_{fn}\\ F_{nf} & 0 \end{array}\right)={\left(\begin{array}{llll} 0 & 0 & F_{13} & F_{14}\\ 0 & 0 & 0 & 0\\ F_{31} & F_{32} & 0 & 0\\ 0 & 0 & 0 & 0 \end{array}\right)}_{8\times 8},$$

where

$$\begin{array}{l} F_{13}={\left(\begin{array}{ll} c_{11}^{fm}\frac{\beta_{mf}}{N_{1}^{m0}}S_{1}^{f0} & c_{12}^{fm}\frac{\beta_{mf}}{N_{2}^{m0}}S_{1}^{f0}\\ c_{21}^{fm}\frac{\beta_{mf}}{N_{1}^{m0}}S_{2}^{f0} & c_{22}^{fm}\frac{\beta_{mf}}{N_{2}^{m0}}S_{2}^{f0} \end{array}\right)}_{2\times 2},\;F_{31}={\left(\begin{array}{ll} c_{11}^{mf}\frac{\beta_{fm}}{N_{1}^{f0}}S_{1}^{m0} & c_{12}^{mf}\frac{\beta_{fm}}{N_{2}^{f0}}S_{1}^{m0}\\ c_{21}^{mf}\frac{\beta_{fm}}{N_{1}^{f0}}S_{2}^{m0} & c_{22}^{mf}\frac{\beta_{fm}}{N_{2}^{f0}}S_{2}^{m0} \end{array}\right)}_{2\times 2},\\ F_{14}=\sigma F_{13},\;F_{32}=\sigma F_{31}, \end{array}$$

and

$$V={\left(\begin{array}{ll} V^{f} & 0\\ 0 & V^{m} \end{array}\right)}_{8\times 8},$$

where

$$\begin{array}{l} V^{g}={\left(\begin{array}{ll} V_{II}^{g} & V_{IC}^{g}\\ V_{CI}^{g} & V_{CC}^{g} \end{array}\right)}_{4\times 4},\cdot V_{IC}^{g}={\left(\begin{array}{ll} -\alpha_{1}^{g} & 0\\ 0 & -\alpha_{2}^{g} \end{array}\right)}_{2\times 2},\cdot V_{II}^{g}={\left(\begin{array}{ll} \gamma_{1}^{g}+a_{1}^{g}+\mu_{1}^{g} & 0\\ -a_{1}^{g} & \gamma_{2}^{g}+\mu_{2}^{g} \end{array}\right)}_{2\times 2},\\ V_{CC}^{g}={\left(\begin{array}{ll} \alpha_{1}^{g}+a_{1}^{g}+\mu_{1}^{g} & 0\\ -a_{1}^{g} & \alpha_{2}^{g}+\mu_{2}^{g} \end{array}\right)}_{2\times 2},\cdot V_{CI}^{g}={\left(\begin{array}{ll} -\left(1-p\right)\gamma_{1}^{g} & 0\\ 0 & -\left(1-p\right)\gamma_{2}^{g} \end{array}\right)}_{2\times 2}, \end{array}$$

with *g* ∈ {*f*, *m*}. Then the next-generation matrix is

$$K\overset{△}{=}FV^{-1}=\left(\begin{array}{ll} 0 & F_{fm}\\ F_{mf} & 0 \end{array}\right)\left(\begin{array}{ll} {\left(V^{f}\right)}^{-1} & 0\\ 0 & {\left(V^{m}\right)}^{-1} \end{array}\right)=\left(\begin{array}{ll} 0 & K_{fm}\\ K_{mf} & 0 \end{array}\right),$$

with

$$\begin{array}{l} {\left(V^{g}\right)}^{-1}=\left(\begin{array}{ll} v_{11}^{g} & v_{12}^{g}\\ v_{21}^{g} & v_{22}^{g} \end{array}\right),\;g\in \left\{f,m\right\},\\ K_{fm}=F_{fm}{\left(V^{m}\right)}^{-1},\;K_{mf}=F_{mf}{\left(V^{f}\right)}^{-1}, \end{array}$$

where

$${\left(V_{II}^{g}\right)}^{-1}={\left(\begin{array}{ll} \frac{1}{M_{1}^{g}} & 0\\ \frac{a_{1}^{g}}{M_{1}^{g}M_{2}^{g}} & \frac{1}{M_{2}^{g}} \end{array}\right)}_{2\times 2},$$

$$\begin{array}{ll} {\left[V_{CC}^{g}-V_{CI}^{g}{\left(V_{II}^{g}\right)}^{-1}V_{IC}^{g}\right]}^{-1} & ={\left(\begin{array}{ll} b_{11}^{g} & 0\\ b_{21}^{g} & b_{22}^{g} \end{array}\right)}_{2\times 2},\\ V_{CC}^{g}-V_{CI}^{g}{\left(V_{II}^{g}\right)}^{-1}V_{IC}^{g} & ={\left(\begin{array}{ll} N_{1}^{g}+\frac{\left(p-1\right)\gamma_{1}^{g}\alpha_{1}^{a}}{M_{1}^{g}} & 0\\ \frac{\left(p-1\right)\gamma_{2}^{g}a_{1}^{g}\alpha_{1}^{g}}{M_{1}^{g}M_{2}^{g}}-a_{1}^{g} & N_{2}^{g}+\frac{\left(p-1\right)\gamma_{2}^{g}\alpha_{2}^{g}}{M_{2}^{g}} \end{array}\right)}_{2\times 2}, \end{array}$$

$$\begin{array}{ll} M_{1}^{g} & =\gamma_{1}^{g}+a_{1}^{g}+\mu_{1}^{g},\\ M_{2}^{g} & =\gamma_{2}^{g}+\mu_{2}^{g},\\ N_{1}^{g} & =\alpha_{1}^{g}+a_{1}^{g}+\mu_{1}^{g},\\ N_{2}^{g} & =\alpha_{2}^{g}+\mu_{2}^{g},\\ b_{11}^{g} & =\frac{1}{N_{1}^{g}+\frac{\left(p-1\right)\gamma_{1}^{g}\alpha_{1}^{g}}{M_{1}^{g}}},\\ b_{21}^{g} & =\frac{\frac{\left(1-p\right)\gamma_{2}^{g}a_{1}^{g}\alpha_{1}^{g}}{M_{1}^{g}M_{2}^{g}}+a_{1}^{g}}{\left[N_{1}^{g}+\frac{\left(p-1\right)\gamma_{1}^{g}\alpha_{1}^{g}}{M_{1}^{g}}\right]\left[N_{2}^{g}+\frac{\left(p-1\right)\gamma_{2}^{g}\alpha_{2}^{g}}{M_{2}^{g}}\right]},\\ b_{22}^{g} & =\frac{1}{N_{2}^{g}+\frac{\left(p-1\right)\gamma_{2}^{g}\alpha_{2}^{g}}{M_{2}^{g}}}, \end{array}$$

$$\begin{array}{ll} v_{11}^{g} & ={\left(\begin{array}{ll} \frac{\alpha_{1}^{g}b_{11}^{g}\left(1-p\right)\gamma_{1}^{g}}{M_{1}^{g}M_{2}^{g}}+\frac{1}{M_{1}^{g}} & 0\\ \left[\frac{\alpha_{1}^{g}a_{1}^{g}b_{11}^{g}}{M_{1}^{g}M_{2}^{g}}+\frac{\alpha_{2}^{g}b_{21}^{g}}{M_{2}^{g}}\right]\frac{1}{M_{1}^{g}}\left(1-p\right)\gamma_{1}^{g}+\frac{a_{1}^{g}\alpha_{2}^{g}b_{22}^{g}\left(1-p\right)\gamma_{2}^{g}}{M_{1}^{g}M_{2}^{g}M_{2}^{g}}+\frac{a_{1}^{g}}{M_{1}^{g}M_{2}^{g}} & \frac{\alpha_{2}^{g}b_{22}^{g}\left(1-p\right)\gamma_{2}^{g}}{M_{2}^{g}M_{2}^{g}}+\frac{1}{M_{2}^{g}} \end{array}\right)}_{2\times 2},\\ v_{12}^{g} & ={\left(\begin{array}{ll} \frac{\alpha_{1}^{g}b_{11}^{g}}{M_{1}^{g}} & 0\\ \frac{a_{1}^{g}\alpha_{1}^{g}b_{11}^{g}}{M_{1}^{g}M_{2}^{g}}+\frac{\alpha_{2}^{g}b_{21}^{g}}{M_{2}^{g}} & \frac{\alpha_{2}^{g}b_{22}^{g}}{M_{2}^{g}} \end{array}\right)}_{2\times 2}, \end{array}$$

$$\begin{array}{l} v_{21}^{g}={\left(\begin{array}{ll} \frac{\left(1-p\right)\gamma_{1}^{g}b_{11}^{g}}{M_{1}^{g}} & 0\\ \frac{\left(1-p\right)\gamma_{1}^{g}b_{21}^{g}}{M_{1}^{g}}+\frac{a_{1}^{g}\left(1-p\right)\gamma_{2}^{g}b_{22}^{g}}{M_{1}^{g}M_{2}^{g}} & \frac{\left(1-p\right)\gamma_{2}^{g}b_{22}^{g}}{M_{2}^{g}} \end{array}\right)}_{2\times 2},\\ v_{22}^{g}={\left(\begin{array}{ll} b_{11}^{g} & 0\\ b_{21}^{g} & b_{22}^{g} \end{array}\right)}_{2\times 2}, \end{array}{\left(V^{g}\right)}^{-1}={\left(\begin{array}{llll} \frac{\alpha_{1}^{g}b_{11}^{g}\left(1-p\right)\gamma_{1}^{g}}{M_{1}^{g}M_{1}^{g}}+\frac{1}{M_{1}^{g}} & 0 & \frac{\alpha_{1}^{g}b_{11}^{g}}{M_{1}^{g}} & 0\\ v_{21} & \frac{\alpha_{2}^{g}b_{22}^{g}\left(1-p\right)\gamma_{2}^{g}}{M_{2}^{g}M_{2}^{g}}+\frac{1}{M_{2}^{g}} & \frac{a_{1}^{g}\alpha_{1}^{g}b_{11}^{g}}{M_{1}^{g}M_{2}^{g}}+\frac{\alpha_{2}^{g}b_{21}^{g}}{M_{2}^{g}} & \frac{\alpha_{2}^{g}b_{22}^{g}}{M_{2}^{g}}\\ \frac{\left(1-p\right)\gamma_{1}^{g}b_{11}^{g}}{M_{1}^{g}} & 0 & b_{11}^{g} & 0\\ \frac{\left(1-p\right)\gamma_{1}^{g}b_{21}^{g}}{M_{1}^{g}}+\frac{a_{1}^{g}\left(1-p\right)\gamma_{2}^{g}b_{22}^{g}}{M_{1}^{g}M_{2}^{g}} & \frac{\left(1-p\right)\gamma_{2}^{g}b_{22}^{g}}{M_{2}^{g}} & b_{21}^{g} & b_{22}^{g} \end{array}\right)}_{2\times 2},$$

with

$$v_{21}=\left[\frac{\alpha_{1}^{g}a_{1}^{g}b_{11}^{g}}{M_{1}^{g}M_{2}^{g}}+\frac{\alpha_{2}^{g}b_{21}^{g}}{M_{2}^{g}}\right]\left(1-p\right)\gamma_{1}^{g}\frac{1}{M_{1}^{g}}+\frac{a_{1}^{g}\alpha_{2}^{g}b_{22}^{g}\left(1-p\right)\gamma_{2}^{g}}{M_{1}^{g}M_{2}^{g}M_{2}^{g}}+\frac{a_{1}^{g}}{M_{1}^{g}M_{2}^{g}}.$$

$$\begin{array}{ll} & K\overset{△}{=}FV^{-1}=\left(\begin{array}{ll} 0 & F_{fm}\\ F_{mf} & 0 \end{array}\right)\left(\begin{array}{ll} {\left(V^{f}\right)}^{-1} & 0\\ 0 & {\left(V^{m}\right)}^{-1} \end{array}\right)\\ & =\left(\begin{array}{llllllll} 0 & 0 & 0 & 0 & f_{1} & f_{2} & \sigma f_{1} & \sigma f_{2}\\ 0 & 0 & 0 & 0 & f_{3} & f_{4} & \sigma f_{3} & \sigma f_{4}\\ 0 & 0 & 0 & 0 & 0 & 0 & 0 & 0\\ 0 & 0 & 0 & 0 & 0 & 0 & 0 & 0\\ f_{5} & f_{6} & \sigma f_{5} & \sigma f_{6} & 0 & 0 & 0 & 0\\ f_{7} & f_{8} & \sigma f_{7} & \sigma f_{8} & 0 & 0 & 0 & 0\\ 0 & 0 & 0 & 0 & 0 & 0 & 0 & 0\\ 0 & 0 & 0 & 0 & 0 & 0 & 0 & 0 \end{array}\right)(\begin{array}{llllllll} v_{1}^{f} & 0 & v_{2}^{f} & 0 & 0 & 0 & 0 & 0\\ v_{3}^{f} & v_{4}^{f} & v_{5}^{f} & v_{6}^{f} & 0 & 0 & 0 & 0\\ v_{7}^{f} & 0 & b_{11}^{f} & 0 & 0 & 0 & 0 & 0\\ v_{8}^{f} & v_{9}^{f} & b_{21}^{f} & b_{22}^{f} & 0 & 0 & 0 & 0\\ 0 & 0 & 0 & 0 & v_{1}^{m} & 0 & v_{2}^{m} & 0\\ 0 & 0 & 0 & 0 & v_{3}^{m} & v_{4}^{m} & v_{5}^{m} & v_{6}^{m}\\ 0 & 0 & 0 & 0 & v_{7}^{m} & 0 & b_{11}^{m} & 0\\ 0 & 0 & 0 & 0 & v_{8}^{m} & v_{9}^{m} & b_{21}^{m} & b_{22}^{m} \end{array})\\ & =\left(\begin{array}{llllllll} 0 & 0 & 0 & 0 & x_{1} & x_{2} & x_{3} & x_{4}\\ 0 & 0 & 0 & 0 & x_{5} & x_{6} & x_{7} & x_{8}\\ 0 & 0 & 0 & 0 & 0 & 0 & 0 & 0\\ 0 & 0 & 0 & 0 & 0 & 0 & 0 & 0\\ x_{9} & x_{10} & x_{11} & x_{12} & 0 & 0 & 0 & 0\\ x_{13} & x_{14} & x_{15} & x_{16} & 0 & 0 & 0 & 0\\ 0 & 0 & 0 & 0 & 0 & 0 & 0 & 0\\ 0 & 0 & 0 & 0 & 0 & 0 & 0 & 0 \end{array}\right), \end{array}$$

where

$$\begin{array}{ll} & f_{1}=c_{11}^{fm}\frac{\beta_{mf}}{N_{1}^{m0}}S_{1}^{f0},\;f_{2}=c_{12}^{fm}\frac{\beta_{mf}}{N_{2}^{m0}}S_{1}^{f0},\;f_{3}=c_{21}^{fm}\frac{\beta_{mf}}{N_{1}^{m0}}S_{2}^{f0},\;f_{4}=c_{22}^{fm}\frac{\beta_{mf}}{N_{2}^{m0}}S_{2}^{f0},\\ & f_{5}=c_{11}^{mf}\frac{\beta_{fm}}{N_{1}^{f0}}S_{1}^{m0},\;f_{6}=c_{12}^{mf}\frac{\beta_{fm}}{N_{2}^{f0}}S_{1}^{m0},\;f_{7}=c_{21}^{mf}\frac{\beta_{fm}}{N_{1}^{f0}}S_{2}^{m0},\;f_{8}=c_{22}^{mf}\frac{\beta_{fm}}{N_{2}^{f0}}S_{2}^{m0},\\ & v_{1}^{g}=\frac{\alpha_{1}^{g}b_{11}^{g}\left(1-p\right)\gamma_{1}^{g}}{M_{1}^{g}M_{1}^{g}}+\frac{1}{M_{1}^{g}},\;v_{2}^{g}=\frac{\alpha_{1}^{g}b_{11}^{g}}{M_{1}^{g}},\;v_{4}^{g}=\frac{\alpha_{2}^{g}b_{22}^{g}\left(1-p\right)\gamma_{2}^{g}}{M_{2}^{g}M_{2}^{g}}+\frac{1}{M_{2}^{g}},\;v_{5}^{g}=\frac{a_{1}^{g}\alpha_{1}^{g}b_{11}^{g}}{M_{1}^{g}M_{2}^{g}}+\frac{\alpha_{2}^{g}b_{21}^{g}}{M_{2}^{g}},\\ & v_{3}^{g}=\left[\frac{\alpha_{1}^{g}a_{1}^{g}b_{11}^{g}}{M_{1}^{g}M_{2}^{g}}+\frac{\alpha_{2}^{g}b_{21}^{g}}{M_{2}^{g}}\right]\left(1-p\right)\gamma_{1}^{g}\frac{1}{M_{1}^{g}}+\frac{a_{1}^{g}\alpha_{2}^{g}b_{22}^{g}\left(1-p\right)\gamma_{2}^{g}}{M_{1}^{g}M_{2}^{g}M_{2}^{g}}+\frac{a_{1}^{g}}{M_{1}^{g}M_{2}^{g}},\;v_{6}^{g}=\frac{\alpha_{2}^{g}b_{22}^{g}}{M_{2}^{g}},\\ & v_{7}^{g}=\frac{\left(1-p\right)\gamma_{1}^{g}b_{11}^{g}}{M_{1}^{g}},\;v_{8}^{g}=\frac{\left(1-p\right)\gamma_{1}^{g}b_{21}^{g}}{M_{1}^{g}}+\frac{a_{1}^{g}\left(1-p\right)\gamma_{2}^{g}b_{22}^{g}}{M_{1}^{g}M_{2}^{g}},\;v_{9}^{g}=\frac{\left(1-p\right)\gamma_{2}^{g}b_{22}^{g}}{M_{2}^{g}},\\ & x_{1}=f_{1}v_{1}^{m}+f_{2}v_{3}^{m}+\sigma f_{1}v_{7}^{m}+\sigma f_{2}v_{8}^{m},\;x_{2}=f_{2}v_{4}^{m}+\sigma f_{2}v_{9}^{m},\;x_{3}=f_{1}v_{2}^{m}+f_{2}v_{5}^{m}+\sigma f_{1}b_{11}^{m}+\sigma f_{2}b_{21}^{m},\\ & x_{4}=f_{2}v_{6}^{m}+\sigma f_{2}b_{22}^{m},\;x_{5}=f_{3}v_{1}^{m}+f_{4}v_{3}^{m}+\sigma f_{3}v_{7}^{m}+\sigma f_{4}v_{8}^{m},\;x_{6}=f_{4}v_{4}^{m}+\sigma f_{4}v_{9}^{m},\\ & x_{7}=f_{3}v_{2}^{m}+f_{4}v_{5}^{m}+\sigma f_{3}b_{11}^{m}+\sigma f_{4}b_{21}^{m},\;x_{8}=f_{4}v_{6}^{m}+\sigma f_{4}b_{22}^{m},\;x_{9}=f_{5}v_{1}^{f}+f_{6}v_{3}^{f}+\sigma f_{5}v_{7}^{f}+\sigma f_{6}v_{8}^{f},\\ & x_{10}=f_{6}v_{4}^{f}+\sigma f_{6}v_{9}^{f},\;x_{11}=f_{5}v_{2}^{f}+f_{6}v_{5}^{f}+\sigma f_{5}b_{11}^{f}+\sigma f_{6}b_{21}^{f},\;x_{12}=f_{6}v_{6}^{f}+\sigma f_{6}b_{22}^{f},\\ & x_{13}=f_{7}v_{1}^{f}+f_{8}v_{3}^{f}+\sigma f_{7}v_{7}^{f}+\sigma f_{8}v_{8}^{f},\;x_{14}=f_{8}v_{4}^{f}+\sigma f_{8}v_{9}^{f},\;x_{15}=f_{7}v_{2}^{f}+f_{8}v_{5}^{f}+\sigma f_{7}b_{11}^{f}+\sigma f_{8}b_{21}^{f},\\ & x_{16}=f_{8}v_{6}^{f}+\sigma f_{8}b_{22}^{f}. \end{array}$$

Furthermore,

(1) $$K_{mf}=\left(\begin{array}{llll} x_{9} & x_{10} & x_{11} & x_{12}\\ x_{13} & x_{14} & x_{15} & x_{16}\\ 0 & 0 & 0 & 0\\ 0 & 0 & 0 & 0 \end{array}\right),\;K_{fm}=\left(\begin{array}{llll} x_{1} & x_{2} & x_{3} & x_{4}\\ x_{5} & x_{6} & x_{7} & x_{8}\\ 0 & 0 & 0 & 0\\ 0 & 0 & 0 & 0 \end{array}\right),$$

(2) $$K_{mf}K_{fm}=\left(\begin{array}{llll} x_{9} & x_{10} & x_{11} & x_{12}\\ x_{13} & x_{14} & x_{15} & x_{16}\\ 0 & 0 & 0 & 0\\ 0 & 0 & 0 & 0 \end{array}\right)\left(\begin{array}{llll} x_{1} & x_{2} & x_{3} & x_{4}\\ x_{5} & x_{6} & x_{7} & x_{8}\\ 0 & 0 & 0 & 0\\ 0 & 0 & 0 & 0 \end{array}\right)$$

(3) $$=\left(\begin{array}{llll} x_{9}x_{1}+x_{10}x_{5} & x_{9}x_{2}+x_{10}x_{6} & x_{9}x_{3}+x_{10}x_{7} & x_{9}x_{4}+x_{10}x_{8}\\ x_{13}x_{1}+x_{14}x_{5} & x_{13}x_{2}+x_{14}x_{6} & x_{13}x_{3}+x_{14}x_{7} & x_{13}x_{4}+x_{14}x_{8}\\ 0 & 0 & 0 & 0\\ 0 & 0 & 0 & 0 \end{array}\right).$$

Let

(4) $$\left\|K_{mf}K_{fm}-\lambda E\right\|=\left|\begin{array}{cccc} x_{9}x_{1}+x_{10}x_{5}-\lambda & x_{9}x_{2}+x_{10}x_{6} & x_{9}x_{3}+x_{10}x_{7} & x_{9}x_{4}+x_{10}x_{8}\\ x_{13}x_{1}+x_{14}x_{5} & x_{13}x_{2}+x_{14}x_{6}-\lambda & x_{13}x_{3}+x_{14}x_{7} & x_{13}x_{4}+x_{14}x_{8}\\ 0 & 0 & -\lambda & 0\\ 0 & 0 & 0 & -\lambda \end{array}\right|$$

(5) $$=\lambda^{2}\left[\left(x_{9}x_{1}+x_{10}x_{5}-\lambda \right)\left(x_{13}x_{2}+x_{14}x_{6}-\lambda \right)-\left(x_{9}x_{2}+x_{10}x_{6}\right)\left(x_{13}x_{1}+x_{14}x_{5}\right)\right]$$

(6) $$=\lambda^{2}\left(\lambda^{2}-b\lambda +c\right)$$

(7) =0,

where

$$b=x_{9}x_{1}+x_{10}x_{5}+x_{13}x_{2}+x_{14}x_{6},\;c=\left(x_{9}x_{1}+x_{10}x_{5}\right)\left(x_{13}x_{2}+x_{14}x_{6}\right)-\left(x_{9}x_{2}+x_{10}x_{6}\right)\left(x_{13}x_{1}+x_{14}x_{5}\right),$$

and hence

$$\lambda_{1}=\lambda_{2}=0,\;\lambda_{3}=\frac{b+\sqrt{b^{2}-4c}}{2},\;\lambda_{4}=\frac{b-\sqrt{b^{2}-4c}}{2}.$$

These are the four eigenvalues of the matrix *K*_*mf*_*K*_*fm*_. Therefore,

$$R_{0}=\rho \left(FV^{-1}\right)=\rho \left(K\right)=\sqrt{\rho \left(K_{mf}K_{fm}\right)}=\sqrt{\frac{b+\sqrt{b^{2}-4c}}{2}}.$$

## Appendix C Proof of Theorem 2

Case 2 Proof of global stability of the disease-free equilibrium (*n* = 2)

Consider the limiting system, that is, when $N^{f0}=\frac{\Lambda^{f}}{\mu^{f}}$ and $N^{m0}=\frac{\Lambda^{m}}{\mu^{m}}$. We define the following Lyapunov function:

$$L=l_{1}I_{1}^{f}+l_{2}I_{2}^{f}+l_{3}C_{1}^{f}+l_{4}C_{2}^{f}+l_{5}I_{1}^{m}+l_{6}I_{2}^{m}+l_{7}C_{1}^{m}+l_{8}C_{2}^{m},\;l_{1},\ldots ,l_{8}>0,$$

where *l* = (*l*_1_, *l*_2_, *l*_3_, *l*_4_, *l*_5_, *l*_6_, *l*_7_, *l*_8_) satisfies the condition $lFV^{-1}=R_{0}l$, that is, $lF=R_{0}lV$. Then

$$\begin{array}{ll} l_{5}f_{1}^{m}+l_{6}f_{3}^{m} & =R_{0}\left[l_{1}\left(\gamma_{1}^{f}+\alpha_{1}^{f}+\mu_{1}^{f}\right)-l_{4}\alpha_{1}^{f}-l_{3}\left(1-p\right)\gamma_{1}^{f}\right],\\ l_{5}f_{2}^{m}+l_{6}f_{4}^{m} & =R_{0}\left[l_{2}\left(\gamma_{2}^{f}+\mu_{2}^{f}\right)-l_{4}\left(1-p\right)\gamma_{2}^{f}\right],\\ l_{5}\sigma f_{1}^{m}+l_{6}\sigma f_{3}^{m} & =R_{0}\left[-l_{4}\alpha_{1}^{f}+l_{3}\left(\alpha_{1}^{f}+a_{1}^{f}+\mu_{1}^{f}\right)-l_{4}a_{1}^{f}\right],\\ l_{5}\sigma f_{2}^{m}+l_{6}\sigma f_{4}^{m} & =R_{0}\left[-l_{2}\alpha_{2}^{f}+l_{4}\left(\alpha_{2}^{f}+\mu_{2}^{f}\right)\right],\\ l_{1}f_{1}^{f}+l_{2}f_{3}^{f} & =R_{0}\left[l_{5}\left(\gamma_{1}^{m}+a_{1}^{m}+\mu_{1}^{m}\right)-l_{6}a_{1}^{m}-l_{7}\left(1-p\right)\gamma_{1}^{m}\right],\\ l_{1}f_{2}^{f}+l_{4}f_{4}^{f} & =R_{0}\left[l_{6}\left(\gamma_{2}^{m}+\mu_{2}^{m}\right)-l_{8}\left(1-p\right)\gamma_{2}^{m}\right],\\ l_{1}\sigma f_{1}^{f}+l_{2}\sigma f_{3}^{f} & =R_{0}\left[-l_{3}a_{1}^{m}+l_{7}\left(\alpha_{1}^{m}+a_{1}^{m}+\mu_{1}^{m}\right)-l_{8}a_{1}^{m}\right],\\ l_{1}\sigma f_{2}^{f}+l_{2}\sigma f_{4}^{f} & =R_{0}\left[-l_{6}a_{2}^{m}+l_{8}\left(\alpha_{2}^{m}+\mu_{2}^{m}\right)\right], \end{array}$$

where

$$f_{1}^{f}\overset{△}{=}c_{11}^{fm}\frac{\beta_{mf}}{N_{1}^{m0}}S_{1}^{f},\;f_{2}^{f}\overset{△}{=}c_{12}^{fm}\frac{\beta_{mf}}{N_{2}^{m0}}S_{1}^{f},\;f_{3}^{f}\overset{△}{=}c_{21}^{fm}\frac{\beta_{mf}}{N_{1}^{m0}}S_{2}^{f},\;f_{4}^{f}\overset{△}{=}c_{22}^{fm}\frac{\beta_{mf}}{N_{2}^{m0}}S_{2}^{f},$$

$$f_{1}^{m}\overset{△}{=}c_{11}^{mf}\frac{\beta_{fm}}{N_{1}^{f0}}S_{1}^{m},\;f_{2}^{m}\overset{△}{=}c_{12}^{mf}\frac{\beta_{fm}}{N_{2}^{f0}}S_{1}^{m},\;f_{3}^{m}\overset{△}{=}c_{21}^{mf}\frac{\beta_{fm}}{N_{1}^{f0}}S_{2}^{m},\;f_{4}^{m}\overset{△}{=}c_{22}^{mf}\frac{\beta_{fm}}{N_{2}^{f0}}S_{2}^{m}.$$

Then

$$\begin{array}{ll} \frac{dL}{dt} & =l_{1}\frac{dI_{1}^{f}}{dt}+l_{2}\frac{dI_{2}^{f}}{dt}+l_{3}\frac{dC_{1}^{f}}{dt}+l_{4}\frac{dC_{2}^{f}}{dt}+l_{5}\frac{dI_{1}^{m}}{dt}+l_{6}\frac{dI_{2}^{m}}{dt}+l_{7}\frac{dC_{1}^{m}}{dt}+l_{8}\frac{dC_{2}^{m}}{dt}\\ & =l_{1}\left[\lambda_{1}^{f}S_{1}^{f}+\alpha_{1}^{f}C_{1}^{f}-\left(\gamma_{1}^{f}+a_{1}^{f}+\mu_{1}^{f}\right)I_{1}^{f}\right]+l_{2}\left[\lambda_{2}^{f}S_{2}^{f}+\alpha_{2}^{f}C_{2}^{f}-\left(\gamma_{2}^{f}+\mu_{2}^{f}\right)I_{2}^{f}+a_{1}^{f}I_{1}^{f}\right]\\ & \;+l_{3}\left[\left(1-p\right)\gamma_{1}^{f}I_{1}^{f}-\left(\alpha_{1}^{f}+a_{1}^{f}+\mu_{1}^{f}\right)C_{1}^{f}\right]+l_{4}\left[\left(1-p\right)\gamma_{2}^{f}I_{2}^{f}-\left(\alpha_{2}^{f}+\mu_{2}^{f}\right)C_{2}^{f}+a_{1}^{f}C_{1}^{f}\right]\\ & \;+l_{5}\left[\lambda_{1}^{m}S_{1}^{m}+\alpha_{1}^{m}C_{1}^{m}-\left(\gamma_{1}^{m}+a_{1}^{m}+\mu_{1}^{m}\right)I_{1}^{m}\right]+l_{6}\left[\lambda_{2}^{m}S_{2}^{m}+\alpha_{2}^{m}C_{2}^{m}-\left(\gamma_{2}^{m}+\mu_{2}^{m}\right)I_{2}^{m}+a_{1}^{m}I_{1}^{m}\right]\\ & \;+l_{7}\left[\left(1-p\right)\gamma_{1}^{m}I_{1}^{m}-\left(\alpha_{1}^{m}+a_{1}^{m}+\mu_{1}^{m}\right)C_{1}^{m}\right]+l_{8}\left[\left(1-p\right)\gamma_{2}^{m}I_{2}^{m}-\left(\alpha_{2}^{m}+\mu_{2}^{m}\right)C_{2}^{m}+a_{1}^{m}C_{1}^{m}\right]. \end{array}$$

Since

$$\lambda_{i}^{g}S_{i}^{g}\leq \lambda_{i}^{g0}S_{i}^{g0},\;g\in \left\{f,m\right\},\;i\in \left\{1,2\right\},$$

it follows that

$$\begin{array}{ll} \frac{dL}{dt} & \leq \left(1-\frac{1}{R_{0}}\right)\left[\left(l_{1}f_{1}^{f}+l_{2}f_{3}^{f}\right)\left(I_{1}^{m}+\sigma C_{1}^{m}\right)+\left(l_{1}f_{2}^{f}+l_{2}f_{4}^{f}\right)\left(I_{2}^{m}+\sigma C_{2}^{m}\right)\right)\\ & \;\left(+\left(l_{5}f_{1}^{m}+l_{6}f_{3}^{m}\right)\left(I_{1}^{f}+\sigma C_{1}^{f}\right)+\left(l_{5}f_{2}^{m}+l_{6}f_{4}^{m}\right)\left(I_{2}^{f}+\sigma C_{2}^{f}\right)\right]. \end{array}$$

When $R_{0}\leq 1$, we have $\frac{dL}{dt}\leq 0$, with equality only at the DFE. Therefore, by the Krasovskii-LaSalle theorem (Martcheva, 2015), the DFE is globally stable when $R_{0}\leq 1$. Using a similar argument, we obtain Theorem 2. Since the DFE exists uniquely and is locally asymptotically stable, it follows that it is globally asymptotically stable.
